# Pentosan polysulfate ameliorates fibrosis and inflammation markers in SV40 MES13 cells by suppressing activation of PI3K/AKT pathway via miR-446a-3p

**DOI:** 10.1186/s12882-022-02732-8

**Published:** 2022-03-15

**Authors:** Liangxiang Xiao, Anqun Chen, Qing Gao, Bo Xu, Xiaodan Guo, Tianjun Guan

**Affiliations:** Department of Nephrology, Zhongshan Hospital of Xiamen University, School of Medicine, Xamen University, No 203, Hubin South Road, Siming district, Xiamen, 361004 Fujian China

**Keywords:** Diabetic nephrology, Advanced glycation end products, microRNA, Pentosan polysulphate sodium, Renal fibrosis

## Abstract

**Background:**

Renal fibrosis is a common outcome of various renal damage, including diabetic nephropathy (DN), the leading cause of end-stage renal disease. Currently, there are no effective therapies for renal fibrosis. The present study aimed to determine whether pentosan polysulphate sodium (PPS), a FDA approved medication for interstitial cystitis, protects diabetic renal fibrosis.

**Methods:**

Cell viability and apoptosis were evaluated in mouse mesangial cells (SV40-MES13) after incubating with the advanced glycation end products (AGEs), which play important roles in the pathogenesis of DN. Western blot and ELISA were performed to determine the expression of transforming growth factor-beta1 (TGF-β1) and fibronectin (FN), two biomarkers of renal fibrosis, as well as interleukin-6 (IL-6) and tumor necrosis factor alpha (TNFα), two biomarkers of inflammation. The miRNA-mRNA regulatory network involved in the phosphatidylinositol 3-kinase (PI3K)/Ser and Thr Kinase (AKT) signalling was investigated by miRNA deep sequencing and validated by RT-PCR and miRNA transfection.

**Results:**

AGEs significantly inhibited cell proliferation and promoted cell apoptosis, which was associated with the overexpression of TGF-β1, FN, IL-6, and TNFα. PPS almost completely reversed AGEs-induced biomarkers of fibrosis and inflammation, and significantly altered the miRNA expression profile in AGEs-treated cells. Notably, the PI3K/AKT signalling was one of the most significantly enriched pathways targeted by PPS-related differentially expressed miRNAs. PPS significantly up-regulated miR-466a-3p, which was shown to target PIK3CA, and mediated the inhibitory effect of PPS on AGEs-induced activation of PI3K/AKT pathway.

**Conclusions:**

The treatment of PPS protected against AGEs-induced toxicity in SV40 MES13 cells via miR-466a-3p-mediated inhibition of PI3K/AKT pathway.

**Supplementary Information:**

The online version contains supplementary material available at 10.1186/s12882-022-02732-8.

## Introduction

Diabetic nephrology (DN), one of the major complications in patients with type 1 and/or type 2 diabetes, is the leading cause of end-stage renal disease (ESRD) globally [1]. Hyperglycemia-induced renal fibrosis, characterized by excessive deposition of extracellular matrix (ECM) and progressive mesangial expansion, plays a critical role in the progression of DN to ESRD [2]. Unfortunately, there are currently no effective therapies for the treatment of diabetic renal fibrosis.

High glucose-induced mesangial ECM production has been implicated in the pathogenesis of DN [3]. The ECM mainly consists of fibronectin (FN), collagens, laminin, and proteoglycans. Among them, FN is one of the first ECM proteins to increase at the early stage of DN [4]. Enhanced FN assembly promotes dysregulated ECM accumulation in DN [3]. Although the mechanism of FN overexpression is not completely understood, the transforming growth factor-beta1 (TGF-β1) has been suggested to play a crucial role [5]. TGF-β1 is also the most important inducer of epithelia-to-mesenchymal transition (EMT) in fibrosis [6]. Nevertheless, massive deposition of FN and excessive secretion of TGF-β1 are two critical indicators of diabetic renal fibrosis.

Advanced glycation end products (AGEs) are a group of heterogeneous compounds formed from nonenzymatic modification of proteins and lipids by sugars under hyperglycemic conditions, and have been implicated in the pathogenesis of DN [7]. AGEs deposit in the mesangium and basement membranes, and directly disrupt matrix-matrix and matrix-cell interactions. AGEs enhance the expression of FN and TGF-β1, and eventually promote the development of DN [8]. AGEs-mediated generation of ROS by activating NADPH oxidase has been suggested to contribute to the overexpression of FN and TGF-β1 [9, 10]. However, it should be noted that the mechanisms for the effects of AGEs on FN and TGF-β1 remain elusive.

Pentosan polysulphate sodium (PPS) is a nonselective anti-inflammatory agent approved by the FDA as the only oral medication for the treatment of interstitial cystitis [11]. PPS has been shown to improve renal function and fibrosis in 5/6 nephrectomized rats [12] and ischemia/reperfusion-injured rats [13]. However, whether PPS protects against diabetic renal fibrosis and the underlying mechanism by which PPS protects against renal injury remain unknown. MicroRNAs (miRNAs or miRs) are small endogenous non-coding RNAs (~ 17-23 nucleotides in length) that induce the mRNA degradation of their target genes by binding to the 3′ non-coding region (3′-UTR) of mRNAs [14]. Studies suggest that miRNAs are involved in the pathogenesis and development of DN [15]. The present study aimed to investigate whether PPS protects against diabetic renal fibrosis in mouse mesangial SV40 MES13 cells, and whether miRNAs play a role in PPS-mediated protection.

## Materials and methods

### Reagents

Dulbecco’s Modified Eagle’s Medium (DMEM) and fetal bovine serum (FBS) were purchased from Gibco (CA, USA). Penicillin/Streptomycin, phosphate-buffered saline (PBS), Lipofectamine 2000 (Lipo2000) were purchased from Invitrogen (CA, USA). All other chemicals were purchased from Sigma-Aldrich Co. (St. Louis, MO).

### Cell culture

Mouse mesangial cells (SV40-MES13) were obtained from the National Infrastructure of Cell Line Resource (Shanghai, China). Cells were grown in DMEM supplemented with 10% FBS and 100 U penicillin/streptomycin under a humidified atmosphere of 5% CO2 at 37 °C. The preparation and dosage of AGEs were referred to previous literature [16]. The dosage of PPS was selected according to previous in vitro studies [17, 18].

### Cell viability assay

The viability of SV40 MES13 cells was determined using a 3-(4,5-dimethylthiazol-2-yl)-2,5-diphenyltetrazolium bromide (MTT) kit (Biosharp, Hefei, China). Briefly, cells were seeded at a density of 2 × 10^4^ cells/mL in 96-well culture plates, and allowed to grow overnight. Cells were incubated in the presence or absence of the test articles for indicted time. After the treatment, 10 μl of MTT reagent was added to each well and incubated at 37 °C for 4 h. The culture media with MTT reagent were then removed, and the formation of formazan crystals were dissolved with 100 μl of detergent reagent. The absorbance of each well was determined using a Tecan Infinite M200 Microplate Reader (Tecan, Männedorf, Zürich, Switzerland) at 540 nm.

### Apoptosis assay

Cells were trypsinized, collected, and washed three times with PBS. The cells were then fixed in 1 mL of 70% ice-cold ethanol overnight at 4 °C. After two washes with PBS and centrifugation for 10 min at 1000 rpm, the supernatant was discarded. Cells were incubated with Annexin V-FITC (5 μl) and PI (5 μl) at room temperature for 30 min in the dark. A cytometric analysis was performed with a flow cytometer (BD Biosciences, San Jose, CA, USA) to measure the apoptosis rates by detecting the relative amount of Annexin V-FITC positive and PI negative cells. Each assay was performed in triplicate.

### Western blot and ELISA analysis

Cells were lysed with RIPA buffer containing protease and phosphatase inhibitors, and the protein concentrations were quantified with the bicinchoninic acid protein assay method. Proteins were separated on 10–15% SDS-PAGE and electroblotted to polyvinylidene fluoride (PVDF) membranes (Bio-Rad, Hercules, CA, USA). Subsequently, the PVDF membranes were blocked and incubated overnight at 4 °C with primary antibodies against FN (1:1000, ab268020, Abcam, Cambridge, MA, USA), TGF-β1 (1:1000, CST3709, Cell Signaling Technology, Inc., Danvers, MA, USA), PIK3CA (1:1000, CST4255S, Cell Signaling Technology, Inc., Danvers, MA, USA), p-PI3K (1:1000, bioworld, AP0152), AKT (1:1000, CST2920, Cell Signaling Technology, Inc., Danvers, MA, USA), p-AKT (1:1000, CST4060, Cell Signaling Technology, Inc., Danvers, MA, USA) and GAPDH (1:5000, ab8245, Abcam, Cambridge, MA, USA). Next, membranes were incubated with the secondary antibody (1:5000) (Santa Cruz Biotechnology, Inc., Santa Cruz, CA, USA) at room temperature for 1 h. The blots were then visualized using a western blot analysis detection system (ECL Plus; GE Healthcare, Princeton, NJ, USA). Interleukin-6 (IL-6) and Tumor necrosis factor alpha (TNFα) in cell lysates were measured using ELISA assays (Cat. No.: CSB-E04639m and CSB-E04741m, Cusabio, Wuhan, China) according to the manufacturer’s instruction.

### miRNA deep sequencing, KEGG enrichment analysis, and interaction analysis of miRNA-mRNA

Total RNA was extracted using TRIzol reagent (Invitrogen, Waltham, MA, USA), and RNA concentrations were determined using a NanoDropTM 2000 (Thermo, MA, US). The integrity of RNA was determined using RNA 6000 Pico kit (Agilent Technologies, Foster City, CA, USA). The miRNA library and deep sequencing were constructed by Forevergen Biosciences Center (Guangzhou, China). miRNAs with |fold changes| ≥ 0.67 and Q-value ≤0.001 were considered significantly differential expression (DE). For more accurate prediction of target genes by DE miRNAs, RNAhybrid and miRanda 3.3a were used to identify the miRNA binding sites. Kyoto Encyclopedia of Genes and Genomes (KEGG) pathway analysis were used to the bioinformatic functions of target gene candidates of differentially expressed miRNAs with DAVID 6.7 software (http://david.abcc.ncifcrf.gov/home.jsp). ACGT101-CORR 1.1 was used to construct the miRNA-mRNA regulatory network for DE miRNAs involved in the PI3K/AKT pathway. The sequencing raw data are available from the NCBI SRA database (accession number: PRJNA769525).

### qRT-PCR (real-time polymerase chain reaction) analysis of miRNAs

Total RNA was extracted using TRIzol reagent (Invitrogen, Waltham, MA, USA), and RNA concentrations were determined using a NanoDropTM 2000 (Thermo, MA, US). qRT-PCR for miRNA was performed using the Stem-Loop miRNA qRT-PCR Primer Set (Forevergen, Guangzhou, China). Data analysis was performed using the 2^−∆∆Ct^ method, and normalized to the expression of U6. All the specific primers were as below.

mmu-miR-19b-3p-RT: GTCGTATCCAGTGCAGGGTCCGAGGTATTCGCACTGGATACGACTCAGTT.

mmu-miR-19b-3p-F:

ACGTCTGTGCAAATCCATGCAA.

mmu-miR-466a-3p-RT:

GTCGTATCCAGTGCAGGGTCCGAGGTATTCGCACTGGATACGACTCTTAT.

mmu-miR-466a-3p-F:

ATCGCTATACATACACGCACAC.

mmu-miR-223-3p-RT:

GTCGTATCCAGTGCAGGGTCCGAGGTATTCGCACTGGATACGACTGGGGT.

mmu-miR-223-3p-F:

ATTCGTGCTGTCAGTTTGTCAA.

mmu-miR-142a-3p-RT:

GTCGTATCCAGTGCAGGGTCCGAGGTATTCGCACTGGATACGACTCCATA.

mmu-miR-142a-3p-F:

ACCTGACTTGTAGTGTTTCCTA.

mmu-miR-19a-3p-RT:

GTCGTATCCAGTGCAGGGTCCGAGGTATTCGCACTGGATACGACtcagtt.

mmu-miR-19a-3p-F:

TGTGCAAATCTATGCAA.

Universe-R 102:

GTGCAGGGTCCGAGGT.

U6-F:

CTCGCTTCGGCAGCACA.

U6-R 142:

AACGCTTCACGAATTTGCGT.

U6-RT:

AACGCTTCACGAATTTGCGT.

### Transfection of miRNAs

The miRNA mimics or inhibitors were purchased from Guangzhou RiboBio (RiboBio, Guangzhou, People’s Republic of China) and transfected into cells using Lipofectamine 2000 (Thermo Fisher Scientific), as recommended by the manufacturer. The cells were transfected with miRNA mimics or inhibitors at a concentration of 100 nM.

### Dual-luciferase assay

Luciferase reporter plasmids containing wild-type (Wt) or mutant (Mut) PIK3CA 3′-UTR sequences (pmirGLO-PIK3CA-Wt or pmirGLO-PIK3CA-Mut) were purchased from Forevergen Biosciences Center (Guangzhou, China). The inserted sequence for pmirGLO-PIK3CA-Wt was 5′-AAGCCGCGAGCCTCCTTGCACAAAATTGATAGGTTTTTTTTTGTGTGTATGTGTGTGTTTGTGTGTGTGTATGTTTAACATTAGTCCATCAGTTGCCGTA-3′. The inserted sequence for pmirGLO-PIK3CA-Mut was 5′- AAGCCGCGAGCCTCCTTGCACAAAATTGATAGGTTTTTTTTTTGTGTATTGGTGGTGTTTGTGTGTGTGTATGTTTAACATTAGTCCATCAGTTGCCGTA − 3′. The plasmids were co-transfected with mmu-miR-466a-3p mimic or mmu-miR-466a-3p inhibitor, respectively, into HEK293T cells using Lipofectamine 2000 (Invitrogen, Carlsbad, CA, USA). The concentrations of plasmids and miRNA mimics were 5 ng/mL and 100 nM, respectively. Cells were harvested at 24 h post-transfection for dual-luciferase activity using the Dual-Glo luciferase assay kit (Promega Corporation, Madison, WI, USA), according to the manufacturer’s instructions.

### Statistical analysis

All experiments were repeated at least three times. The statistical analysis was performed using SPSS19.0 statistical software. Data are presented as mean ± SEM. Student’s *t-*test (unpaired, two-tailed) analyses were applied to evaluate the differences between two groups. ANOVA was applied to calculate the differences among various groups. *P* < 0.05 was considered statistically significant.

## Results

### PPS attenuated the cytotoxicity of AGEs in SV40 MES13 cells

AGEs play a critical role in the development and progression of DN, and have been used to induce renal fibrosis and inflammation [16]. In the present study, the addition of 200 μg/mL AGEs to mouse kidney mesangial cells SV40 MES13 did not alter the cell proliferation at 24 h and 48 h, but significantly inhibited cell proliferation (16%↓) at 72 h (Supplemental Fig. S1A). Flow cytometry analysis showed that AGEs treatment for 24 h increased cell apoptosis (Supplemental Fig. S1B). The protein levels of fibronectin (FN) and TGF-β1, which initiate and participate in the progression of diabetic renal fibrosis, were markedly increased by AGEs treatment for 24 h (Supplemental Fig. S1C). ELISA showed that IL-6 and TNFα, two inflammatory biomarkers, were significantly increased in SV40 MES13 cells after incubating with 200 μg/ml AGEs for 24 h (Supplemental Fig. S1D). These results suggest that AGEs are able to induce fibrosis and inflammation in SV40 MES13 cells.

**Fig. 1:**
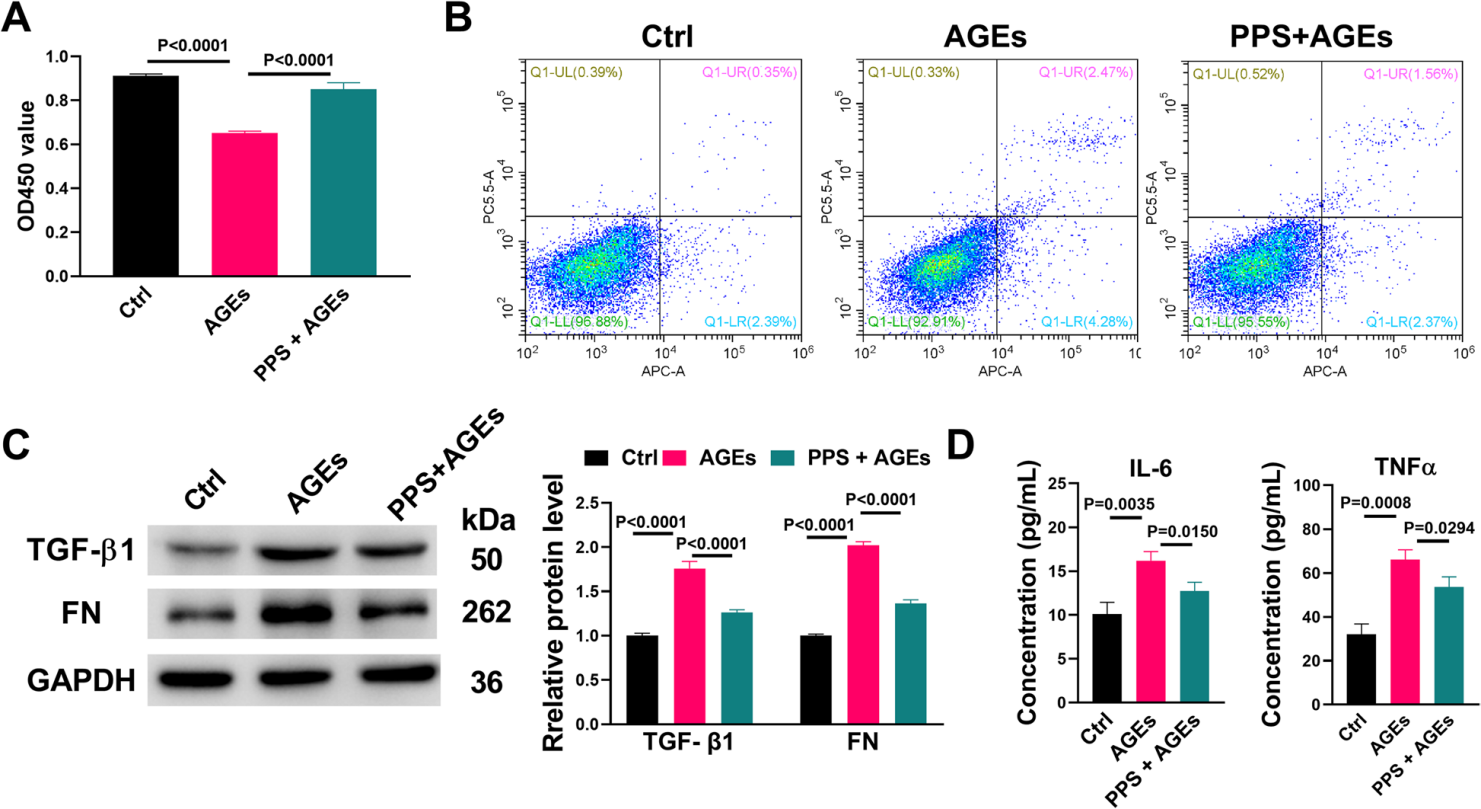
PPS attenuated the cytotoxicity of AGEs in SV40 MES13 cells. **A** SV40 RES13 cells were treated with 200 μg/mL AGEs or AGEs+PPS for 72 h, and the cell viability was determined by MTT assay. **B** Flow cytometry was used to determine the apoptotic rate of SV40 RES13 cells treated with AGEs for 72 h. **C** Representative western blots of TGF-β1 and FN proteins in SV40 RES13 cells treated with AGEs or AGEs+PPS for 72 h, as well as statistical analyses of western blots. **D** The protein levels of IL-6 and TNFα were determined by ELISA assay in SV40 RES13 cells treated with AGEs or AGEs+PPS for 72 h. The results were analyzed by one-way ANOVA and are expressed as mean ± SEM. *N* = 3

To evaluate the effect of PPS on AGEs-induced toxicity, SV40 MES13 cells were treated with AGEs in the presence or absence of PPS for 72 h. As shown in Fig. 1A, PPS significantly reversed the inhibitory effect of AGEs on cell proliferation in SV40 MES13 cells. Flow cytometry analysis revealed that PPS also inhibited the apoptosis induced by AGEs in SV40 MES13 cells (Fig. 1B). Western blot and ELISA analysis demonstrated that PPS attenuated AGEs-induced up-regulation of fibrosis biomarkers (FN and TGF- β1) and inflammatory biomarkers (IL-6 and TNFα), respectively (Fig. 1C and D). These results suggest that PPS protects against AGEs-induced cytotoxicity in SV40 MES13 cells.

### PPS altered the miRNA expression profile and inhibited the activation of PI3K/AKT signalling in AGEs-treated SV40 MES13 cells

**Fig. 2:**
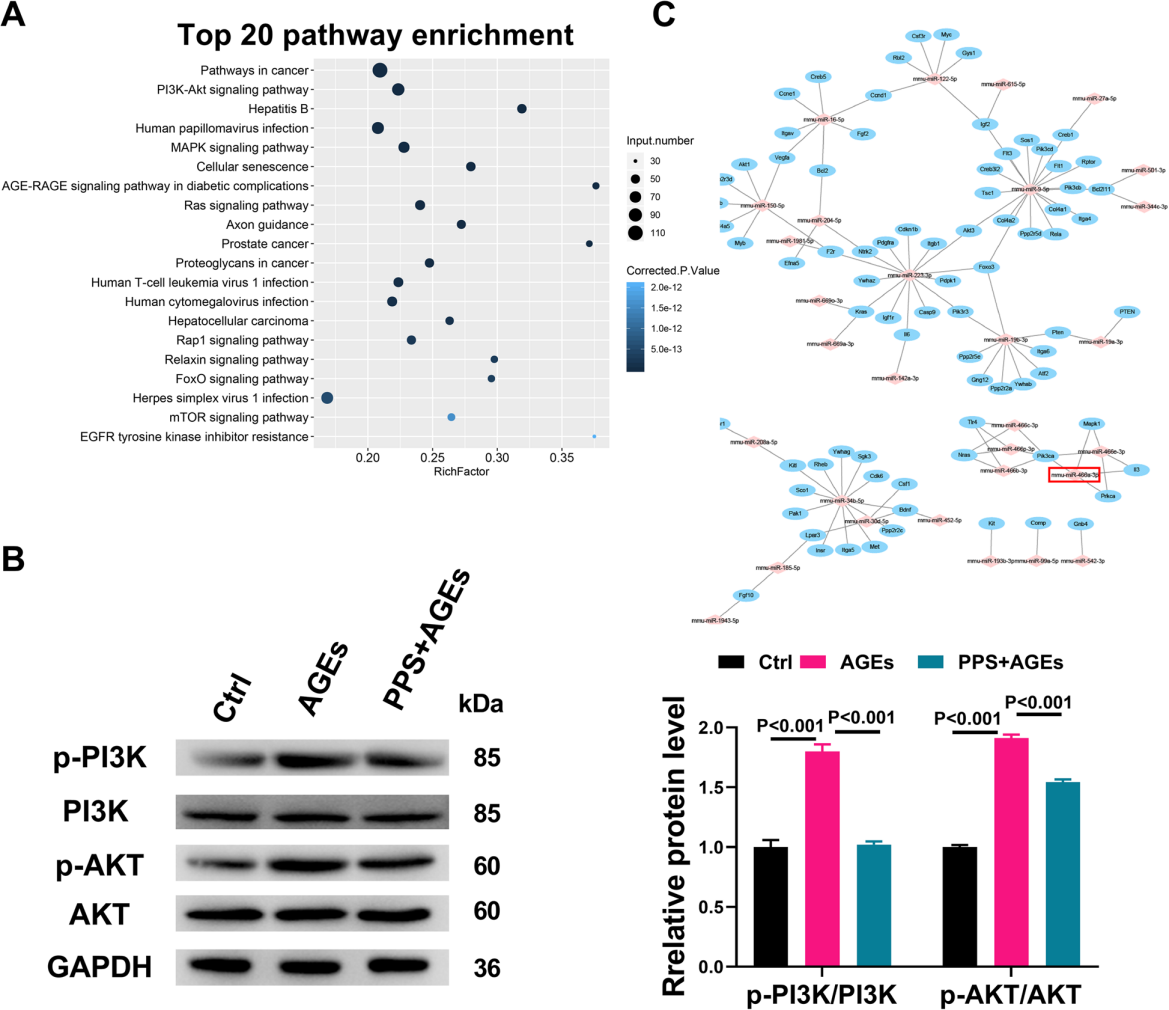
PPS inhibited the PI3K/AKT signaling in AGEs-treated SV40 MES13 cells. **A** Top 20 enriched KEGG pathways of the putative target genes of the differentially expressed miRNAs induced by PPS co-treatment in AGEs-treated SV40 RES13 cells. **B** Representative western blots of p-PI3K, PI3K, p-AKT, and AKT in SV40 RES13 cells treated with AGEs in the presence or absence of PPS for 24 h, as well as statistical analyses of western blots. **C** Network map of miRNA-mRNA interactions of the differentially expressed miRNAs involved in the PI3K/AKT pathway. Red square frame highlights mmu-miR-466a-3p

To investigate whether miRNAs were involved in the mechanisms underlying the protective effect of PPS on AGEs-induced cytotoxicity, we performed miRNA sequencing and demonstrated that PPS markedly altered the miRNA expression profile in AGEs-treated SV40 MES13 cells (Supplemental Fig. S2). Differential expression levels of miRNAs showed that PPS significantly up-regulated 44 miRNAs and down-regulated 26 miRNAs in AGEs-treated SV40 MES13 cells with fold changes more than 0.67 (Supplemental Table S1). GO analysis revealed the diverse biological roles of the targets of differentially expressed miRNAs (Supplemental Fig. S3). The differentially expressed miRNAs were also involved in various pathways in the KEGG database (Fig. 2A). Among them, the PI3K/AKT signalling pathway, which plays a crucial role in the pathogenesis of fibrosis by regulating many factors upstream and downstream, was the second most significantly enriched pathway. Western blot demonstrated that AGEs significantly increased the phosphorylation of PI3K and AKT proteins in SV40 MES13 cells, whereas such effect was almost completely reversed by co-treatment with PPS (Fig. 2B). Next, we identified 115 target genes of the 30 differentially expressed miRNAs involved in the PI3K/AKT signalling pathway that might participate in PPS-mediated protection against AGEs-induced cytotoxicity in SV40 MES13 cells (Fig. 2C). Taken together, PPS alters the miRNA expression profile and inhibits the activation of PI3K/AKT signalling in AGEs-treated SV40 MES13 cells.

### miR-466a-3p mediated the protective effect of PPS on AGEs-induced fibrosis and inflammation

**Fig. 3:**
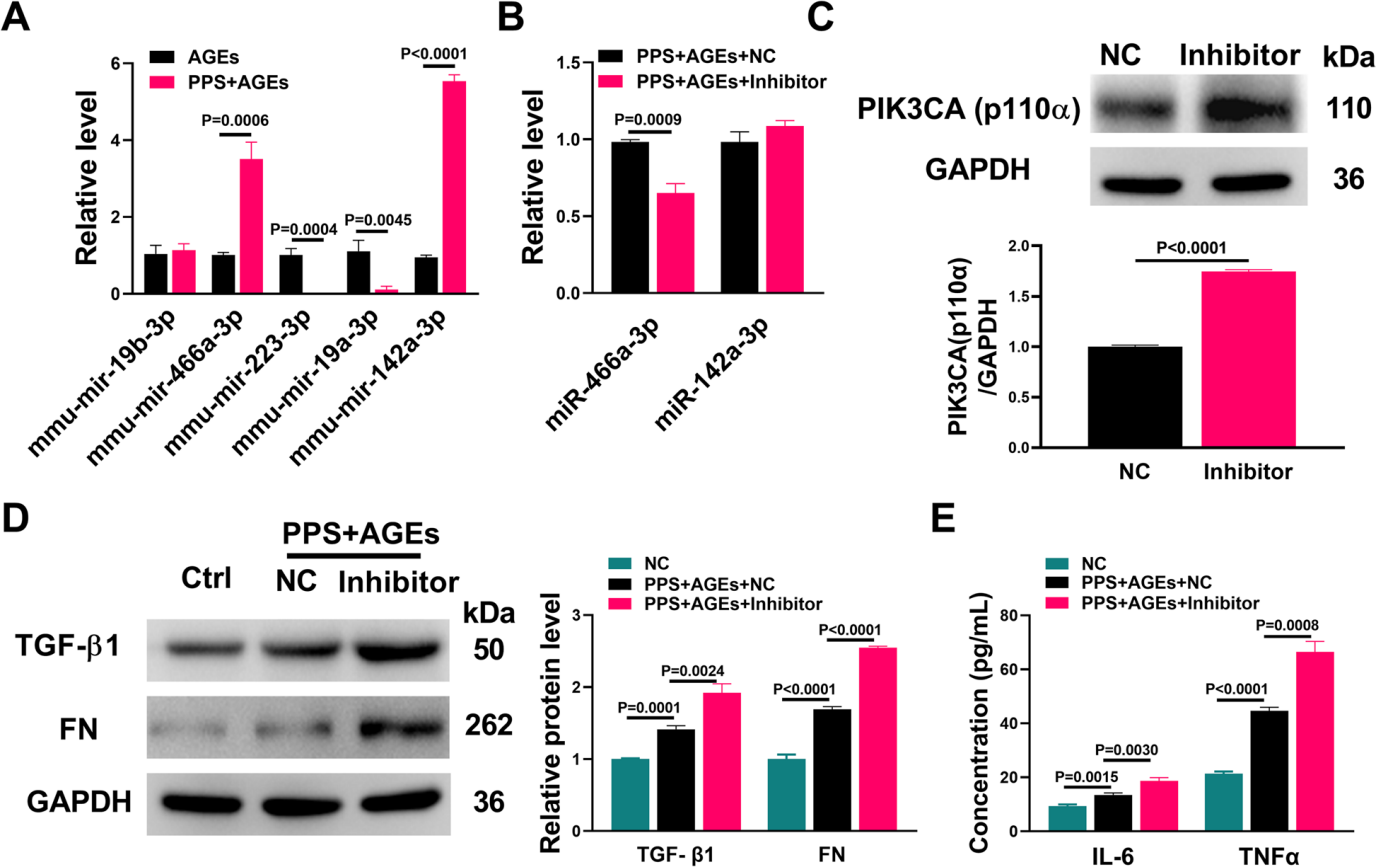
miR-466a-3p mediated the protective effect of PPS on AGEs-induced fibrosis and inflammation. **A** Expression of miRNAs was determined by qRT-PCR in SV40 RES13 cells treated with AGEs or PPS + AGEs for 24 h. **B** The miR-446-3p negative control (NC) or inhibitor was transfected into SV40 RES13 cells treated with AGEs and PPS. The expression of miRNAs was determined by qRT-PCR. **C** Western blot analyses of PIK3CA. The miR-446-3p negative control (NC) or inhibitor was transfected into SV40 RES13 cells for 48 h. The protein level of PIK3CA was determined by western blot. **D** Western blot analyses of TGF-β1 and FN. The miR-446-3p negative control (NC) or inhibitor was transfected into SV40 RES13 cells treated with AGEs and PPS for 48 h. The protein levels of TGF- β1 and FN were determined by western blot. **E** The miR-446-3p negative control or inhibitor was transfected into SV40 RES13 cells treated with AGEs and PPS for 48 h. The protein levels of IL-6 and TNFα were determined by ELISA assay. All the experiments were performed in triplicate, and the results were analyzed by one-way ANOVA or Student’s *t-*test (unpaired, two-tailed). The data are expressed as mean ± SEM.

Next, we used RT-PCR to validate 5 selected differentially expressed miRNAs in the PI3K/AKT signalling pathway. Among them, mmu-miR-466a-3p (2.5 fold↑) and mmu-mir-142a-3p (4.5 fold↑) were significantly up-regulated, whereas mmu-mir-223-3p and mmu-mir-19a-3p were down-regulated more than 99.9% by PPS in AGEs-treated SV40 MES13 cells (Fig. 3A). It should be noted that miR-466a-3p targets PIK3CA and miR-142a-3p targets IL-6, respectively. We then synthesized inhibitors of these two miRNAs and demonstrated that the expression of miR-466a-3p, but not miR-142a-3p, was able to be decreased by the miRNA inhibitors in SV40 MES13 cells co-treated with PPS and AGEs (Fig. 3B). Additionally, overexpression of the miR-466a-3p inhibitor significantly increased the protein expression of PI3KCA, the target gene of miR-466a-3p (Fig. 3C). Western blot and ELISA results demonstrated that miR-466a-3p inhibitor increased the protein levels of fibrosis (FN and TGF-β1) and inflammatory (IL-6 and TNFα) biomarkers in SV40 MES13 cells co-treated with PPS and AGEs (Fig. 3D and E). These results suggest that miR-466a-3p mediates the protective effect of PPS on AGEs-induced cytotoxicity in SV40 MES13 cells.

### miR446a-3p inhibited PI3K/AKT signalling pathway via PI3Kca in AGEs-treated SV40 MES13 cells

**Fig. 4:**
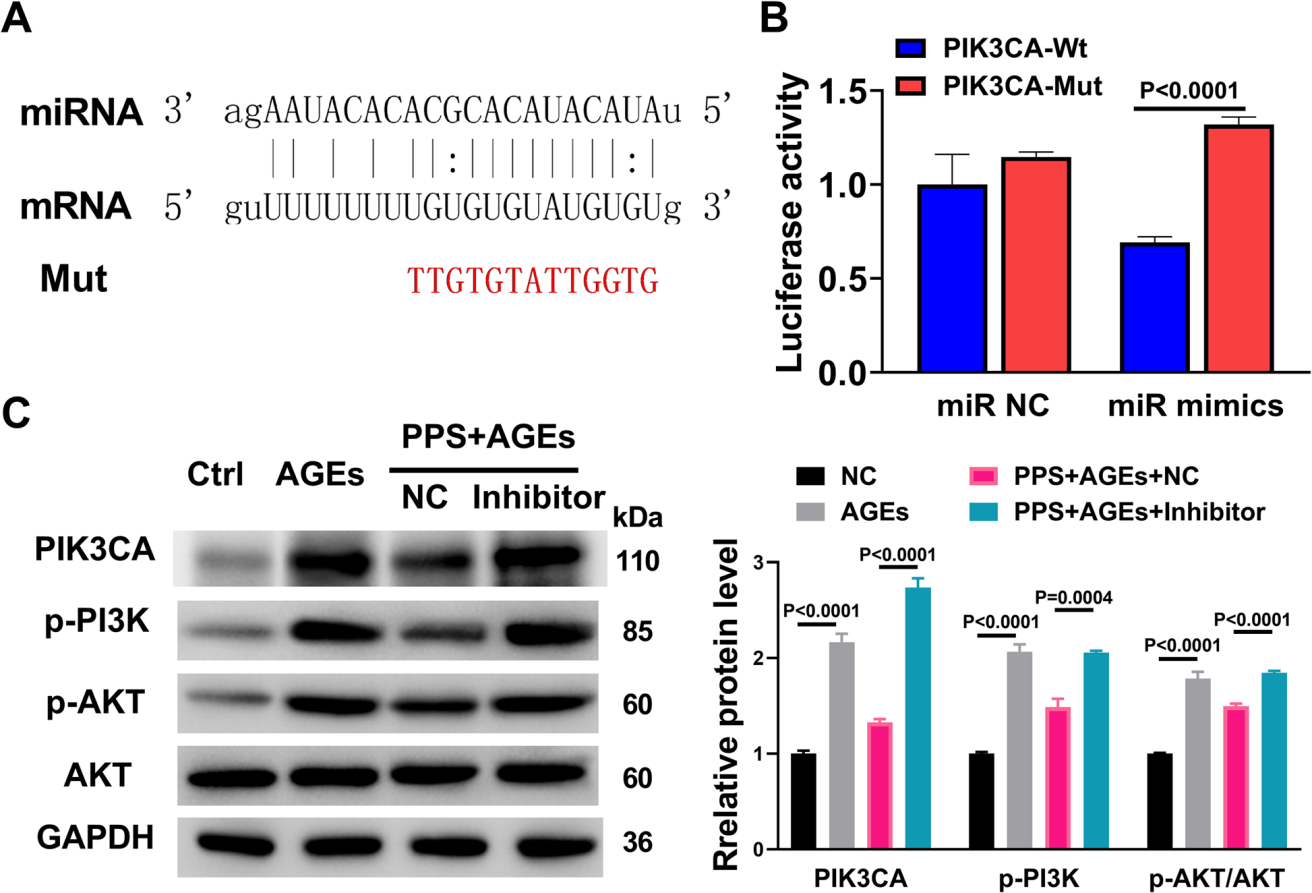
miR-466a-3p inhibited PI3K/AKT signaling pathway via PIK3CA in AGEs-treated SV40 MES13 cells. **A** Schematic graph of the putative binding sites of miR-466a-3p in the PIK3CA 3′-UTR. Mut 3′-UTR indicates the PIK3CA 3′-UTR with a mutation in the miR-466a-3p binding sites. **B** miR-466a-3p mimics downregulated the activity of a luciferase reporter containing wild-type PIK3CA 3′-UTR but not of a reporter containing mutant PIK3CA 3′-UTR. **C** Forty-eight hours after miR-466a-3p control and inhibitor transfection of SV40 RES13 cells treated with AGEs and PPS, the protein levels of PIK3CA, p-PI3K, pAKT, and AKT were determined by Western blot analysis. GAPDH was used as the internal control. All the experiments were performed in triplicate. The results were analyzed by one-way ANOVA (more than two groups) or Student’s *t-*test (unpaired, two-tailed, two groups). The data are expressed as mean ± SEM.

In view of the important role of PI3K/AKT signalling in the development of renal fibrosis, we next determined the mechanism by which miR-466a-3p mediated the protective effect of PPS on AGEs-induced cytotoxicity. Bioinformatics analysis predicted the binding sites of miR-446a-4p in mRNA of PIK3CA, and the mutant PIK3CA (Mut) was constructed according to predicted sequence (Fig. 4A). Luciferase assay was performed to compare the effect of miR-466a-3p mimics on the expression of PI3Kca. Co-expression of miR-466a-3p mimics with PIK3CA-Mut significantly increased luciferase activity compared with co-expression of miR-466a-3p with PIK3CA-Wt in HEK293T cells, suggesting a binding between miR-466a-3p and PIK3CA (Fig. 4B). Western blot analysis showed that the inhibitory effect of PPS on AGEs-induced up-regulation of PIK3CA as well as phosphorylated PI3K and AKT was almost completely reversed by miR-466a-3p inhibitors (Fig. 4C). These results suggest that miR-466a-3p interacts with PIK3CA and inhibits the activation of PI3K/AKT signalling pathway in AGEs-treated SV40 MES13 cells.

## Discussion

PPS is a mixture of sulphated polyanions that have been widely used for the treatment of interstitial cystitis, an inflammatory-like disease. Studies suggests a protective effect of PPS on renal function in various models, but it remains unknown whether PPS protects against AGEs-induced DN. In the present study, we showed that AGEs were able to inhibit proliferation and promote apoptosis of SV40 RES13 cells, which was associated with elevated fibrosis (TGF-β1 and FN) and inflammation (IL-6 and TNFα) biomarkers. TGF-β1 is a central mediator of tubulointerstitial fibrosis by inducing the occurrence of EMT and fibrogenesis [19]. Our results showed that AGEs-treated SV40 RES13 cells could be used as in vitro model of renal fibrosis. More importantly, we showed that PPS almost completely reversed AGEs-induced biomarkers of fibrosis and inflammation in SV40 RES13 cells.

Several mechanisms have been proposed for the protective effect of PPS on renal function. Wu et al. reported that PPS inhibits NF-kB and inflammatory responses in mice with severe diabetic nephropathy [18]. Chen et al. demonstrated that PPS inhibits high glucose-induced activation of p38 MAPK pathway in human renal proximal tubular epithelial cells (HK-2) [20]. To best of our knowledge, we reported here for the first time that miRNAs play a crucial role in PPS-mediated protective effects on renal function. Several miRNAs have been associated with the development and progression of DN [15, 21, 22]. The present study showed that PPS markedly altered the miRNA expression profile in AGEs-treated SV40 RES13 cells. Notably, PI3K/AKT signalling and MAPK signalling were among top 5 most significantly enriched pathways targeted by PPS-related differentially expressed miRNAs. Importantly, both PI3K/AKT and MAPK pathways have been shown to be involved in the mechanisms of fibrosis, and have been suggested to be potential targets for antifibrosis therapy [23–25].

The present study identified the protective effect of miR-466a-3p on AGEs-induced toxicity in SV40 RES13 cells. miR-466a-3p was shown to negatively target the mRNA of PIK3CA, which codes for the p110α isoform of class-IA PI3K. The inhibitor of miR-466a-3p almost completely reversed the effect of PPS on AGEs-induced activation of PI3K/AKT pathway as well as overexpression of FN, TGF-β1, IL-6 and TNFα. This suggests that miR-466a-3p-PI3K/AKT axis mediates the protective effect of PPS on renal function. Future studies are needed to further elucidate the role of miR-466a-3p in cell proliferation, apoptosis, and extracellular matrix in mesangial cells.

Although AGEs have been implicated in the pathogenesis of diabetic DN, the mechanism by which it enhances FN and TGF-β1 is not completely clear [9]. AGEs-induced ROS could increase cytokines and growth factors, and thus lead to overexpression of FN and TGF-β1 in diabetic renal fibrosis [10]. It should be noted that the ROS induced by AGEs could further stimulate the formation of new AGEs, resulting in a positive feedback loop [9, 26]. The present study revealed that AGEs markedly activated the PI3K/AKT pathway in SV40 RES13 cells, and inhibition of this pathway by PPS almost completely reversed the toxicity of AGEs.

## Conclusions

To conclude, PPS protects against AGEs-induced toxicity in SV40 RES13 cells by altering the miRNAs. miR-466a-3p targets the mRNA of PIK3CA, and mediates the protective effect of PPS via inhibition of PI3K/AKT pathway. It should be noted that the present study is limited by focusing on only one miRNA involved in the PI3K/AKT pathway. Future studies are needed to elucidate the role of miRNAs involved in other pathways.

## Supplementary Information

**Additional file 1.**

## Data Availability

The sequencing raw data are available from the NCBI SRA database under the accession number PRJNA769525. The other data are available upon request.

## Abbreviations

AGEs: Advanced glycation end products
AKT: Ser and Thr Kinase
DN: Diabetic nephrology
ECM: Extracellular matrix
FN: Fibronectin
IL-6: Interleukin-6
PI3K: Phosphatidylinositol 3-kinase
PPS: Pentosan polysulphate sodium
TGF-β1: Transforming growth factor-beta1
TNFα: Tumor necrosis factor alpha
