# Supplementary material for: Pentosan polysulfate ameliorates fibrosis and inflammation markers in SV40 MES13 cells by suppressing activation of PI3K/AKT pathway via miR-446a-3p
Source: BMC Nephrol. 2022 Mar 15;23:105. doi: 10.1186/s12882-022-02732-8 (PMC8925175; doi:10.1186/s12882-022-02732-8)
Supplement: Supplementary file 1 — Additional file 1. [file 12882_2022_2732_MOESM1_ESM.zip › original western blot images.pptx]

## Slide 1
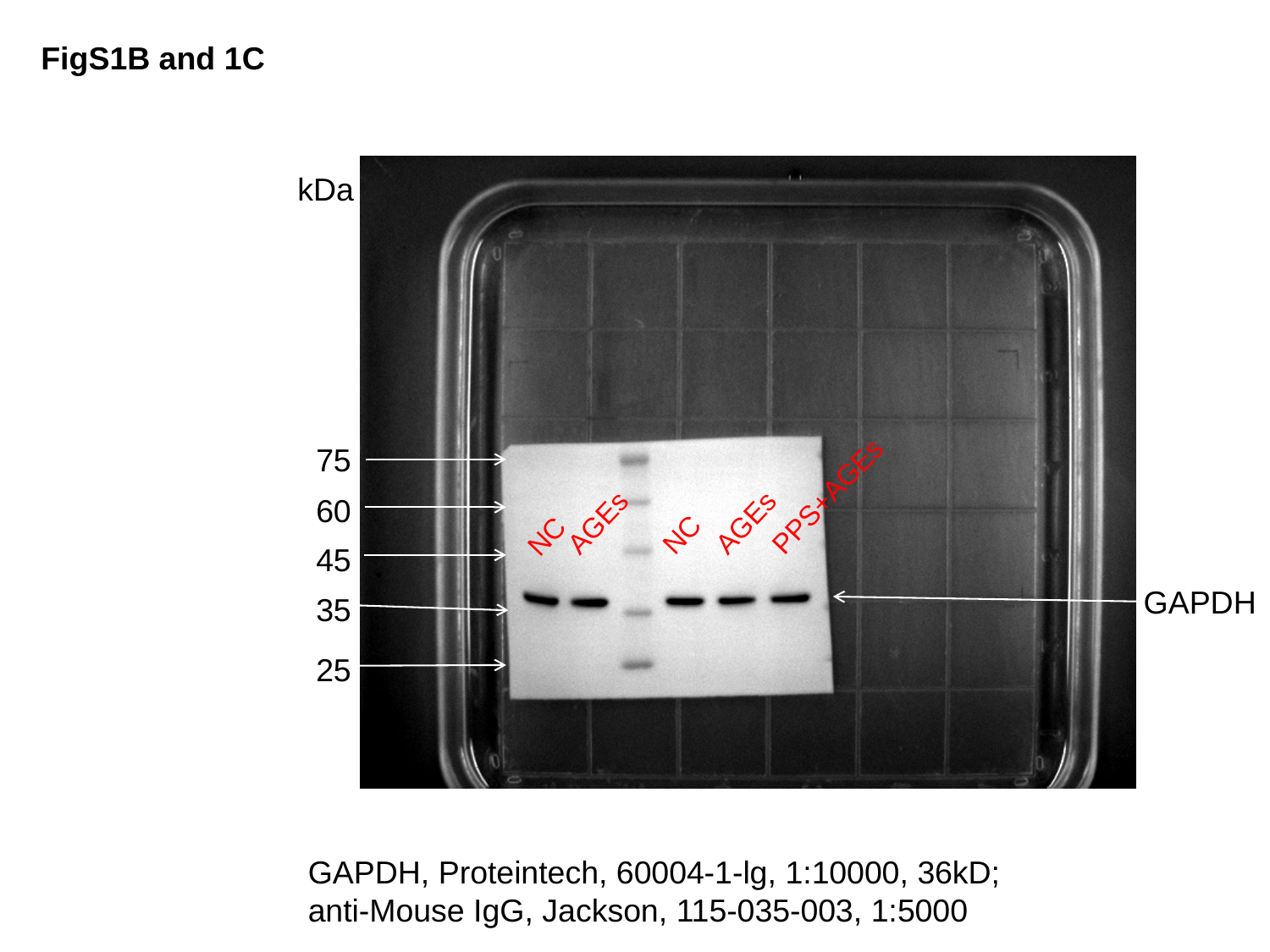

FigS1B and 1C
kDa
PPS+AGEs
NC
AGEs
AGEs
NC
75
60
45
35
25
GAPDH
GAPDH, Proteintech, 60004-1-lg, 1:10000, 36kD;
anti-Mouse IgG, Jackson, 115-035-003, 1:5000

## Slide 2
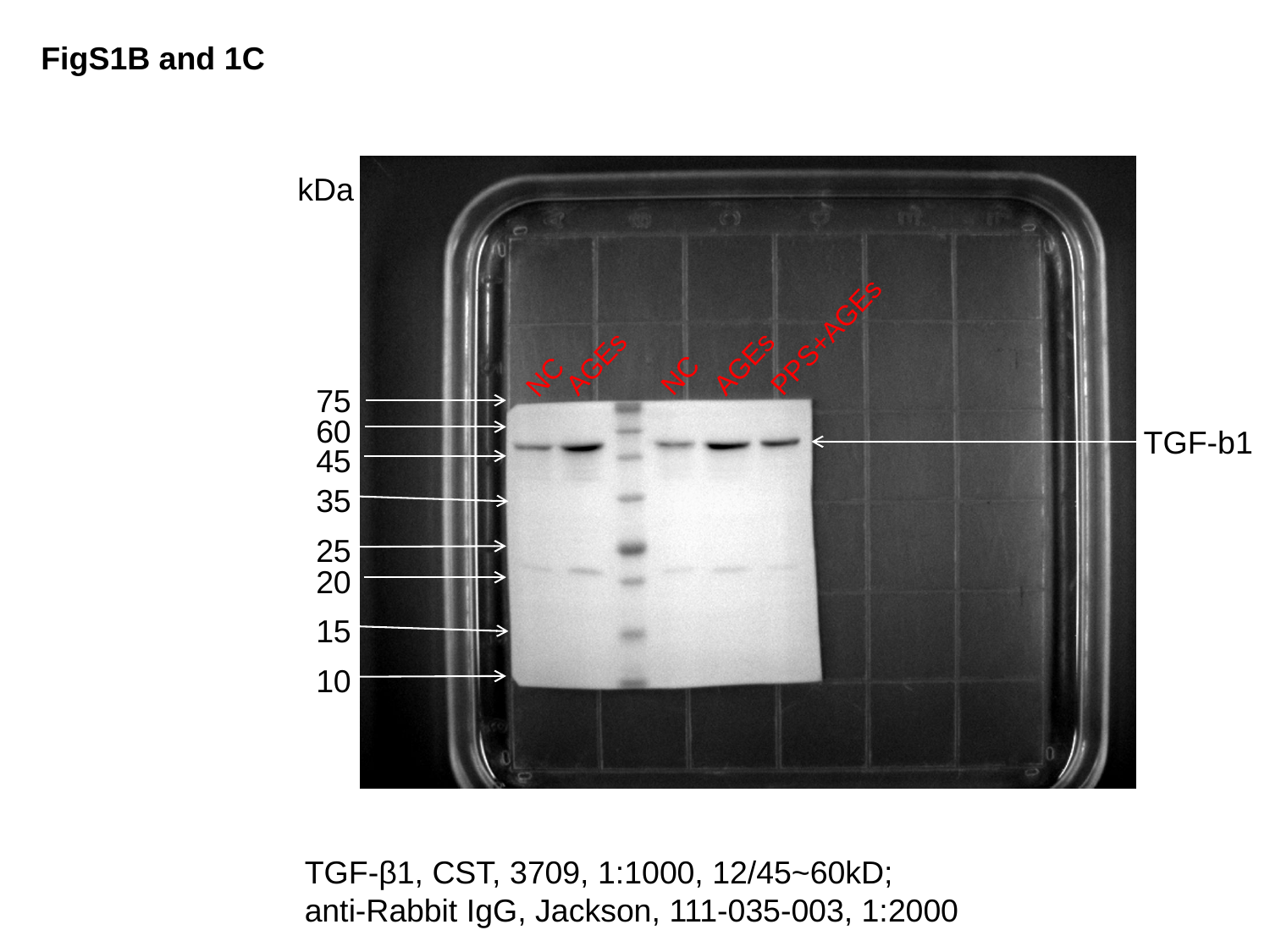

FigS1B and 1C
kDa
PPS+AGEs
NC
AGEs
AGEs
NC
75
60
45
35
25
20
15
10
TGF-b1
TGF-β1, CST, 3709, 1:1000, 12/45~60kD;
anti-Rabbit IgG, Jackson, 111-035-003, 1:2000

## Slide 3
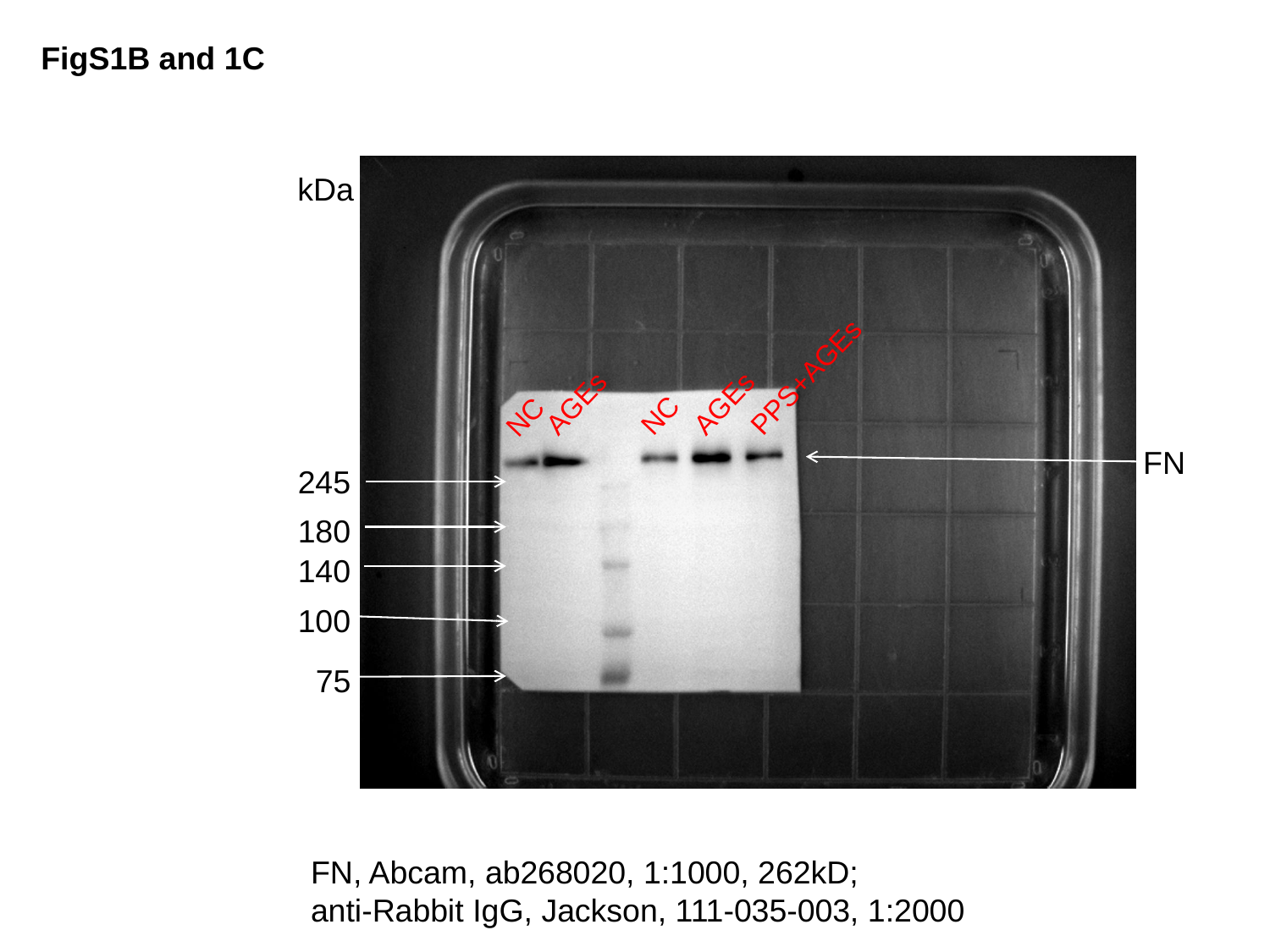

FigS1B and 1C
kDa
PPS+AGEs
NC
AGEs
AGEs
NC
FN
245
180
140
100
75
FN, Abcam, ab268020, 1:1000, 262kD;
anti-Rabbit IgG, Jackson, 111-035-003, 1:2000

## Slide 4
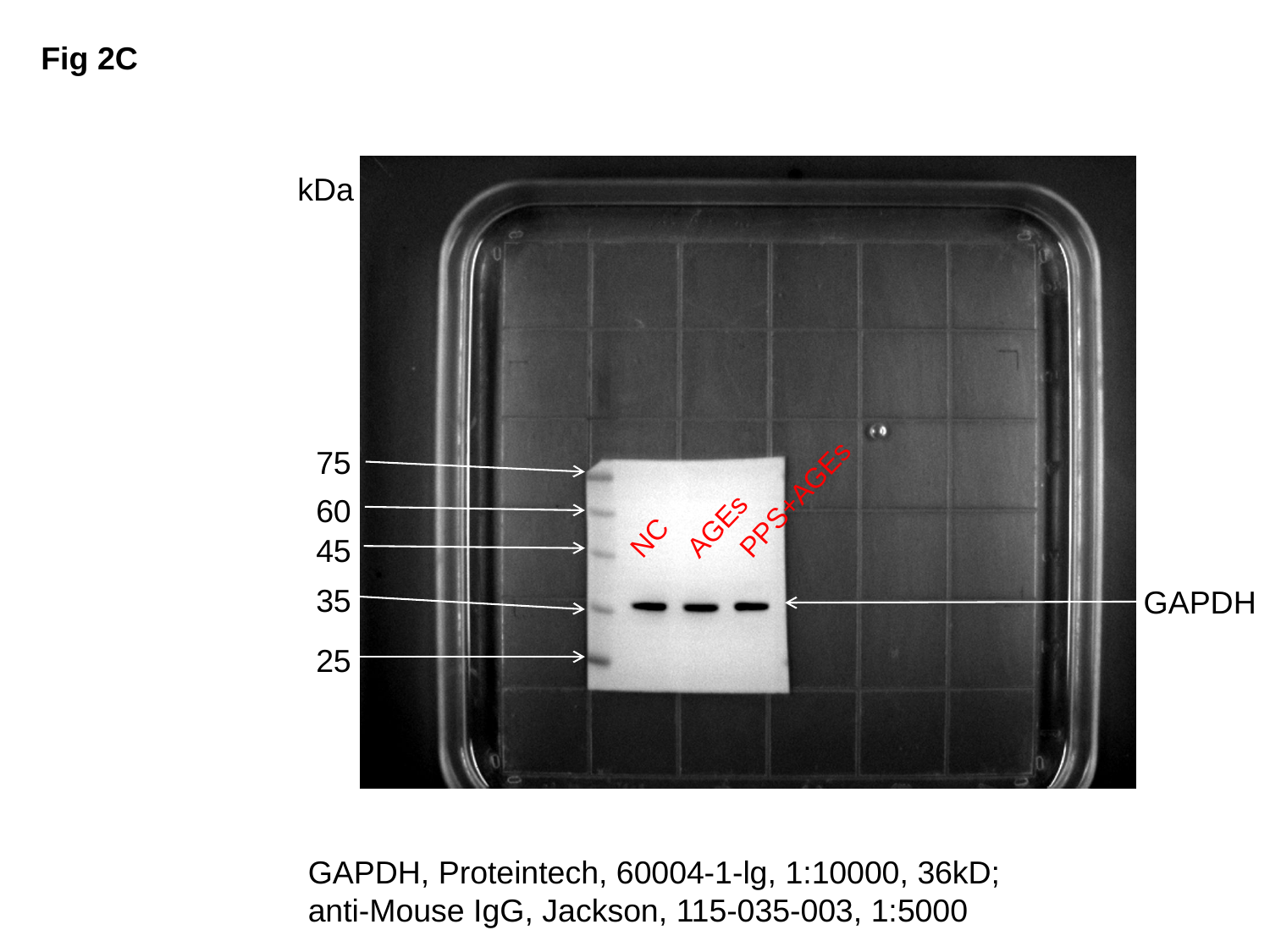

Fig 2C
kDa
AGEs
PPS+AGEs
NC
75
60
45
35
25
GAPDH
GAPDH, Proteintech, 60004-1-lg, 1:10000, 36kD;
anti-Mouse IgG, Jackson, 115-035-003, 1:5000

## Slide 5
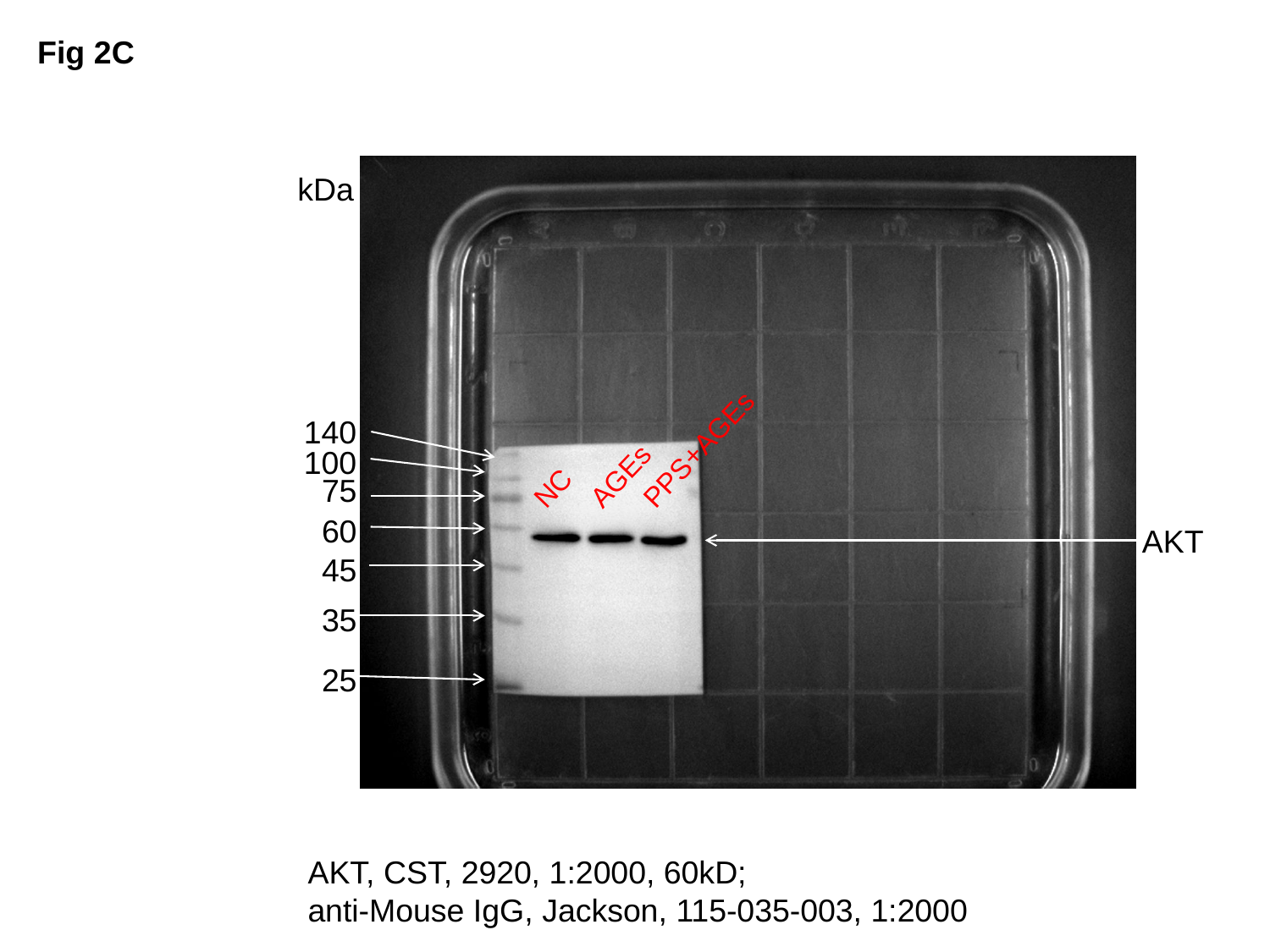

Fig 2C
kDa
AGEs
PPS+AGEs
NC
140
100
75
60
45
35
25
AKT
AKT, CST, 2920, 1:2000, 60kD;
anti-Mouse IgG, Jackson, 115-035-003, 1:2000

## Slide 6
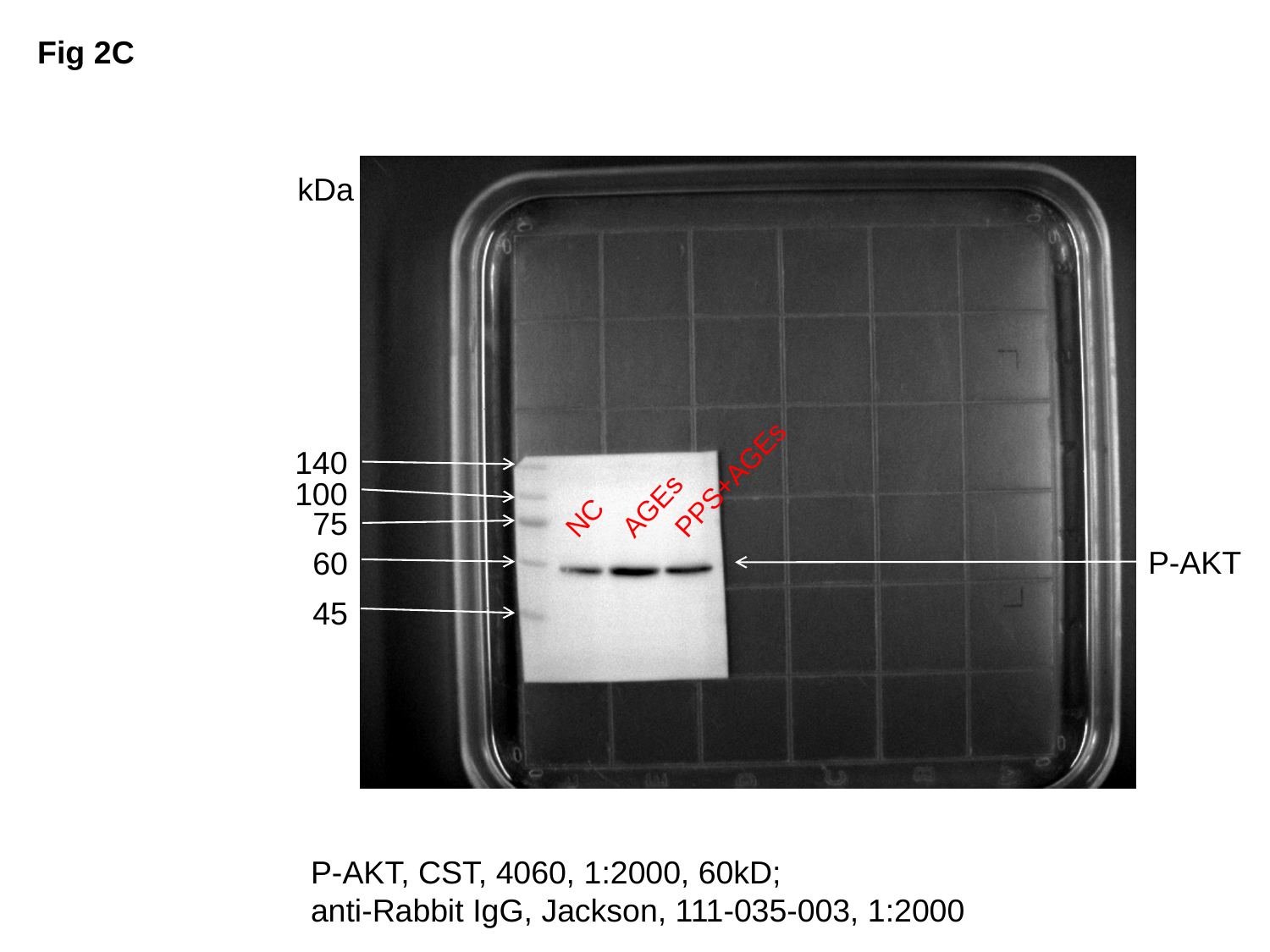

Fig 2C
kDa
AGEs
PPS+AGEs
NC
140
100
75
60
45
P-AKT
P-AKT, CST, 4060, 1:2000, 60kD;
anti-Rabbit IgG, Jackson, 111-035-003, 1:2000

## Slide 7
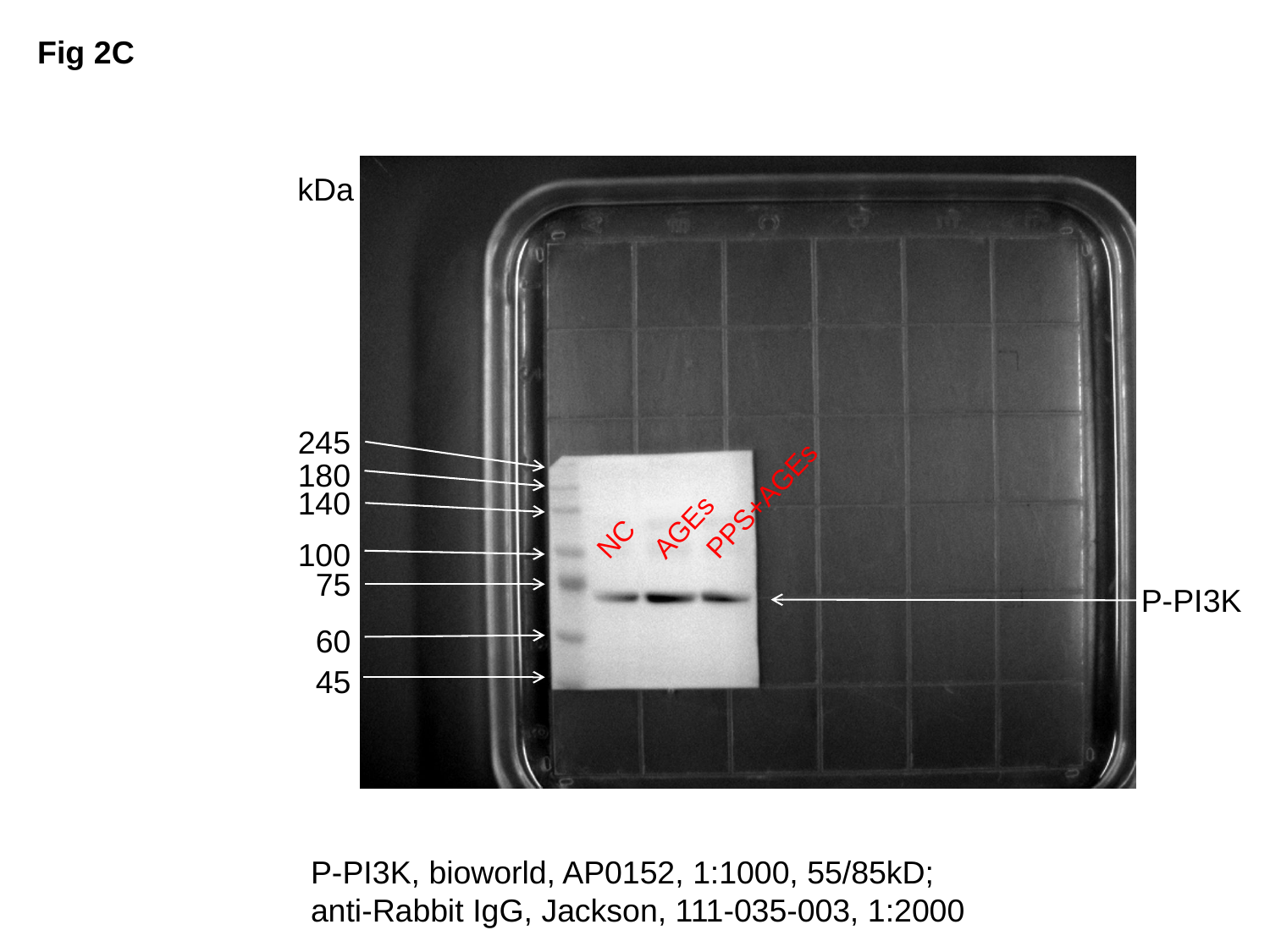

Fig 2C
kDa
AGEs
PPS+AGEs
NC
245
180
140
100
75
60
45
P-PI3K
P-PI3K, bioworld, AP0152, 1:1000, 55/85kD;
anti-Rabbit IgG, Jackson, 111-035-003, 1:2000

## Slide 8
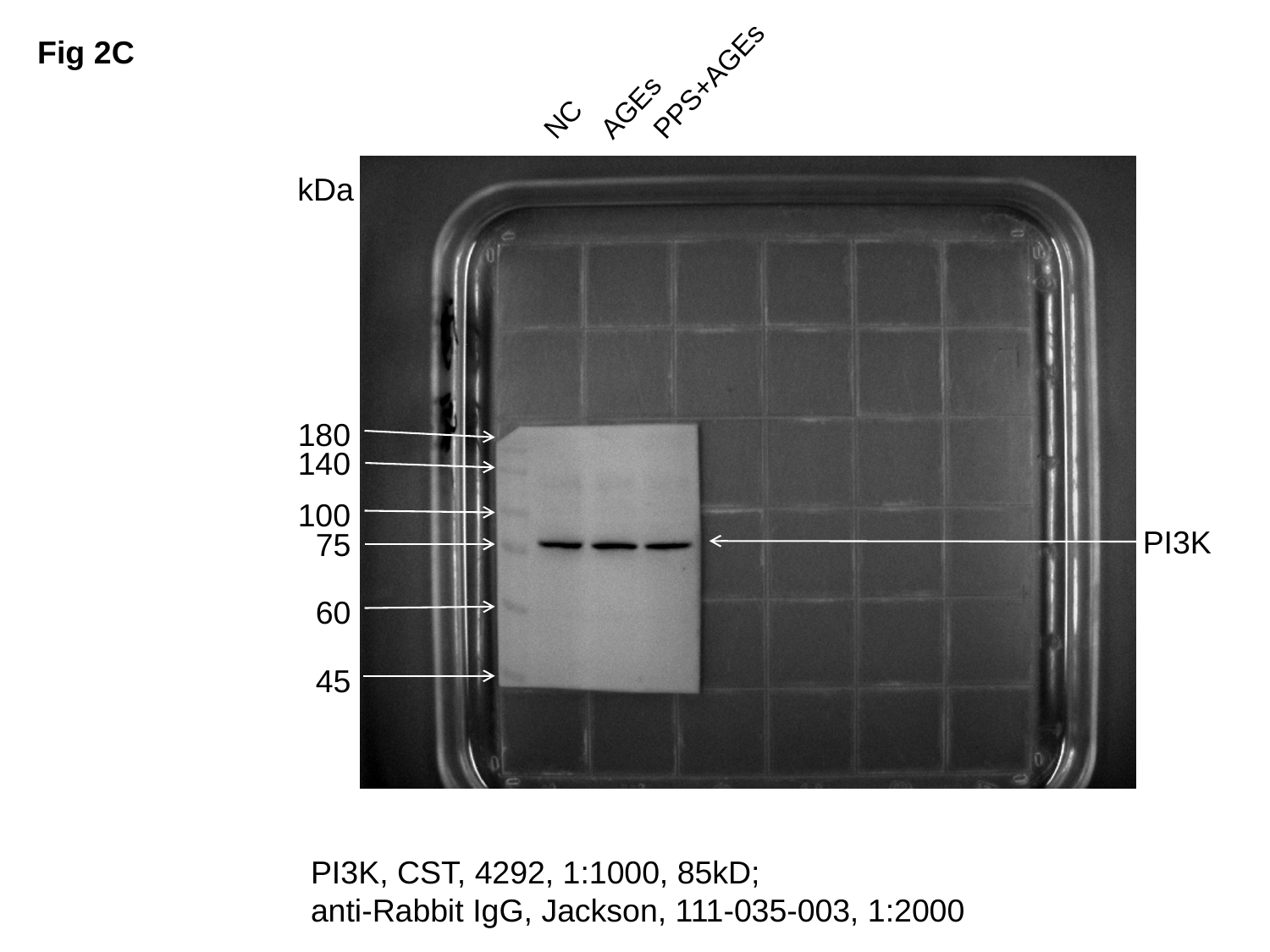

AGEs
PPS+AGEs
NC
Fig 2C
kDa
180
140
100
75
60
45
PI3K
PI3K, CST, 4292, 1:1000, 85kD;
anti-Rabbit IgG, Jackson, 111-035-003, 1:2000

## Slide 9
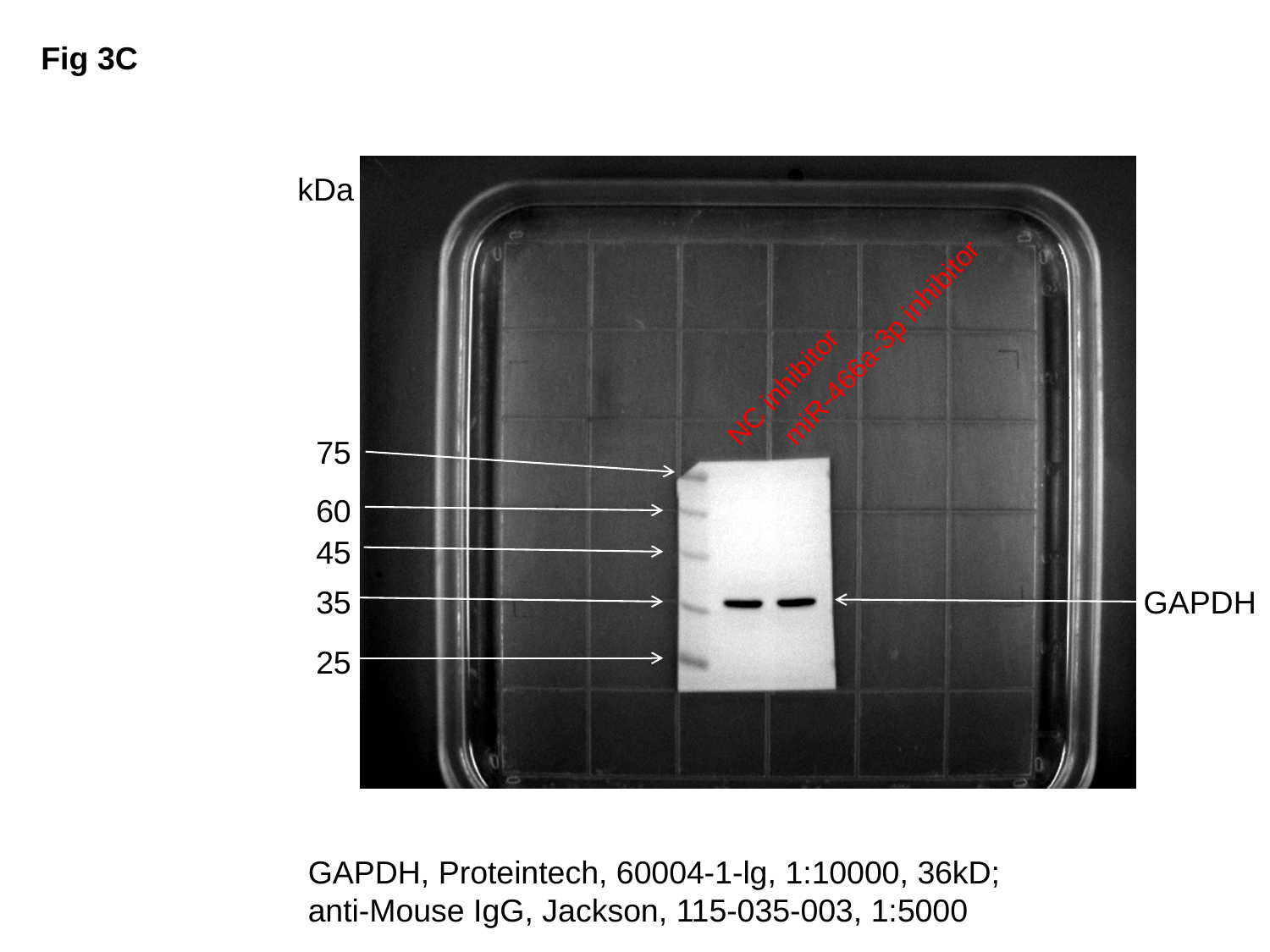

Fig 3C
kDa
miR-466a-3p inhibitor
NC inhibitor
75
60
45
35
25
GAPDH
GAPDH, Proteintech, 60004-1-lg, 1:10000, 36kD;
anti-Mouse IgG, Jackson, 115-035-003, 1:5000

## Slide 10
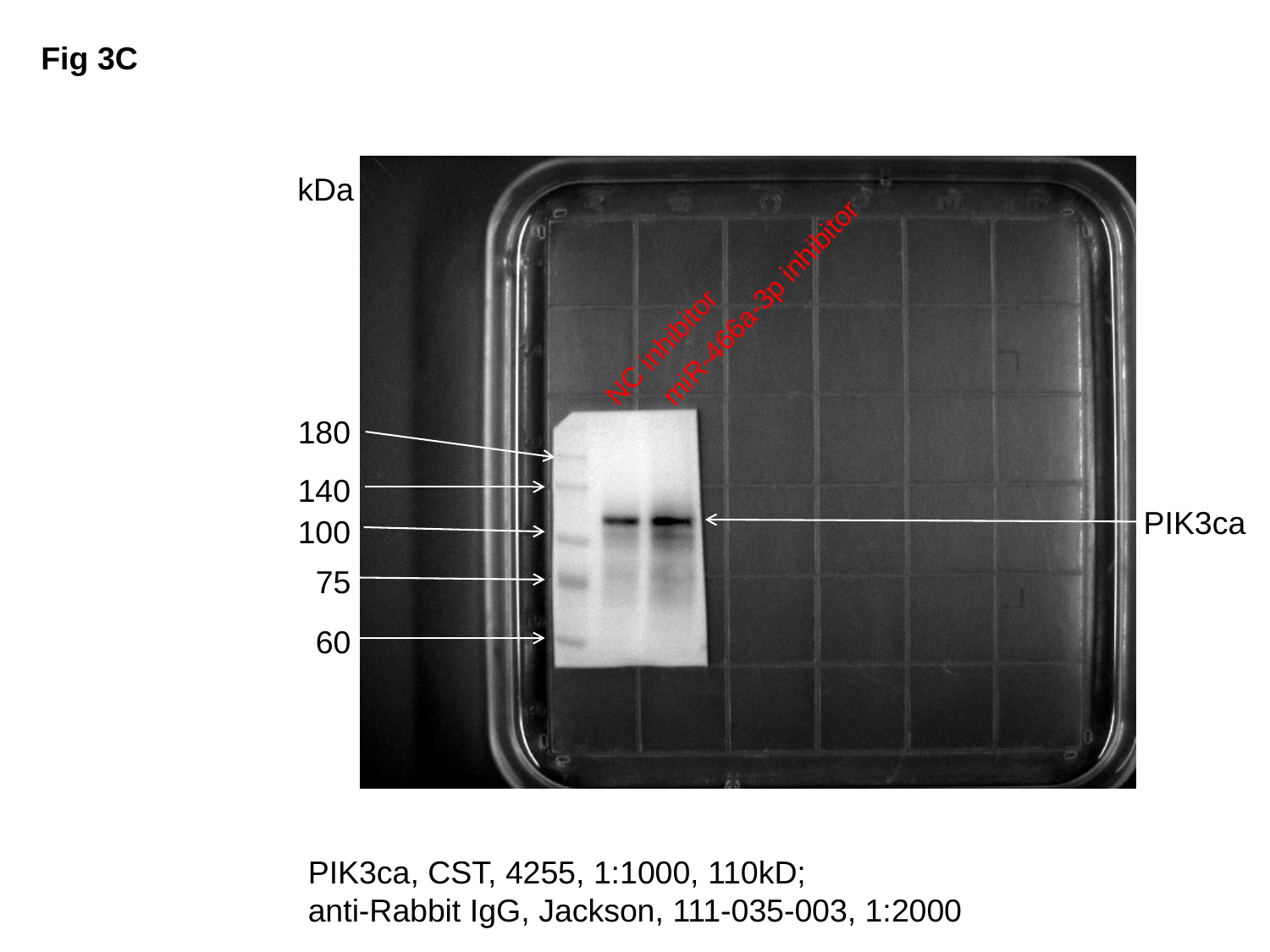

Fig 3C
miR-466a-3p inhibitor
NC inhibitor
kDa
180
140
100
75
60
PIK3ca
PIK3ca, CST, 4255, 1:1000, 110kD;
anti-Rabbit IgG, Jackson, 111-035-003, 1:2000

## Slide 11
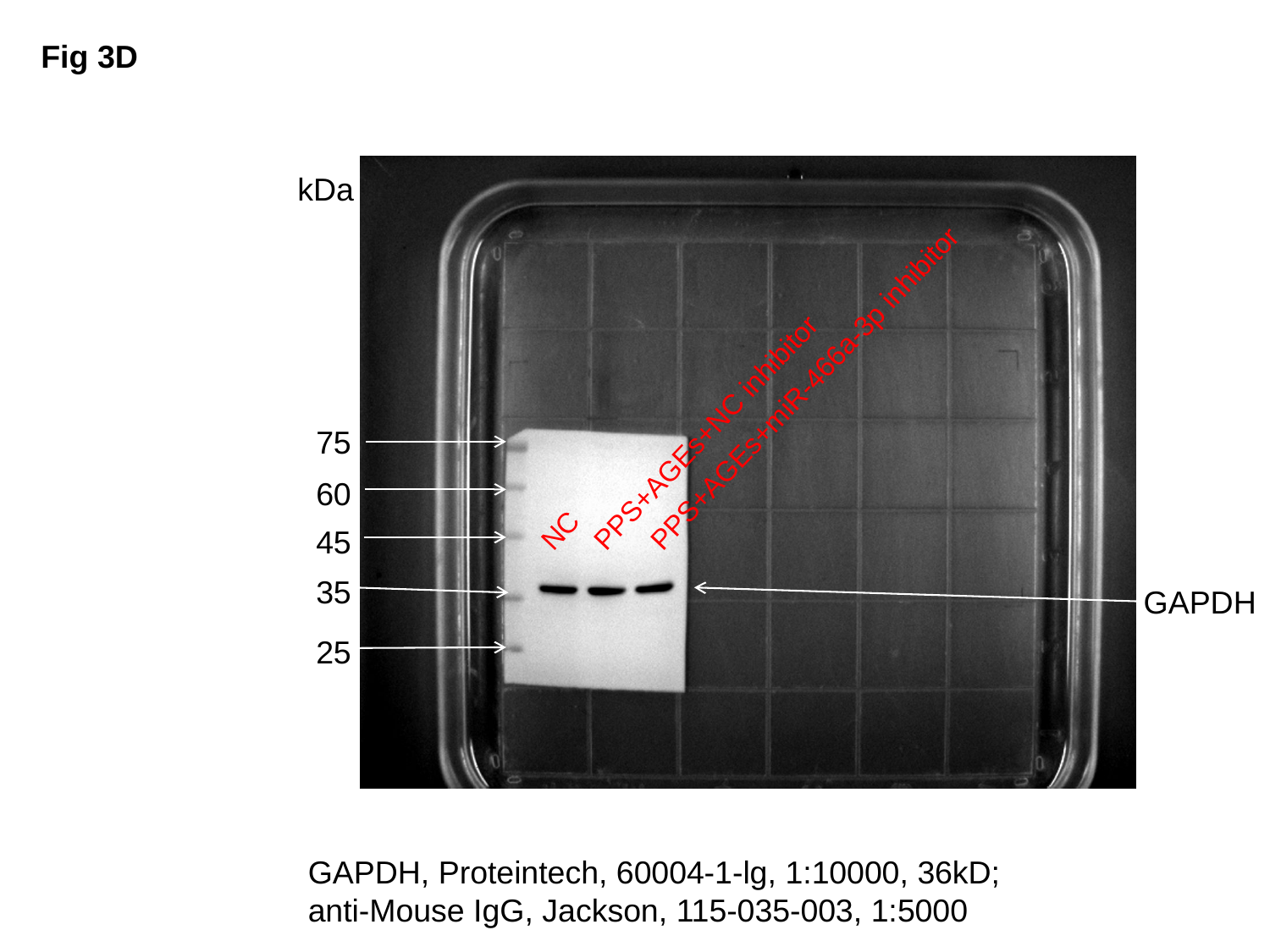

Fig 3D
PPS+AGEs+miR-466a-3p inhibitor
PPS+AGEs+NC inhibitor
NC
kDa
75
60
45
35
25
GAPDH
GAPDH, Proteintech, 60004-1-lg, 1:10000, 36kD;
anti-Mouse IgG, Jackson, 115-035-003, 1:5000

## Slide 12
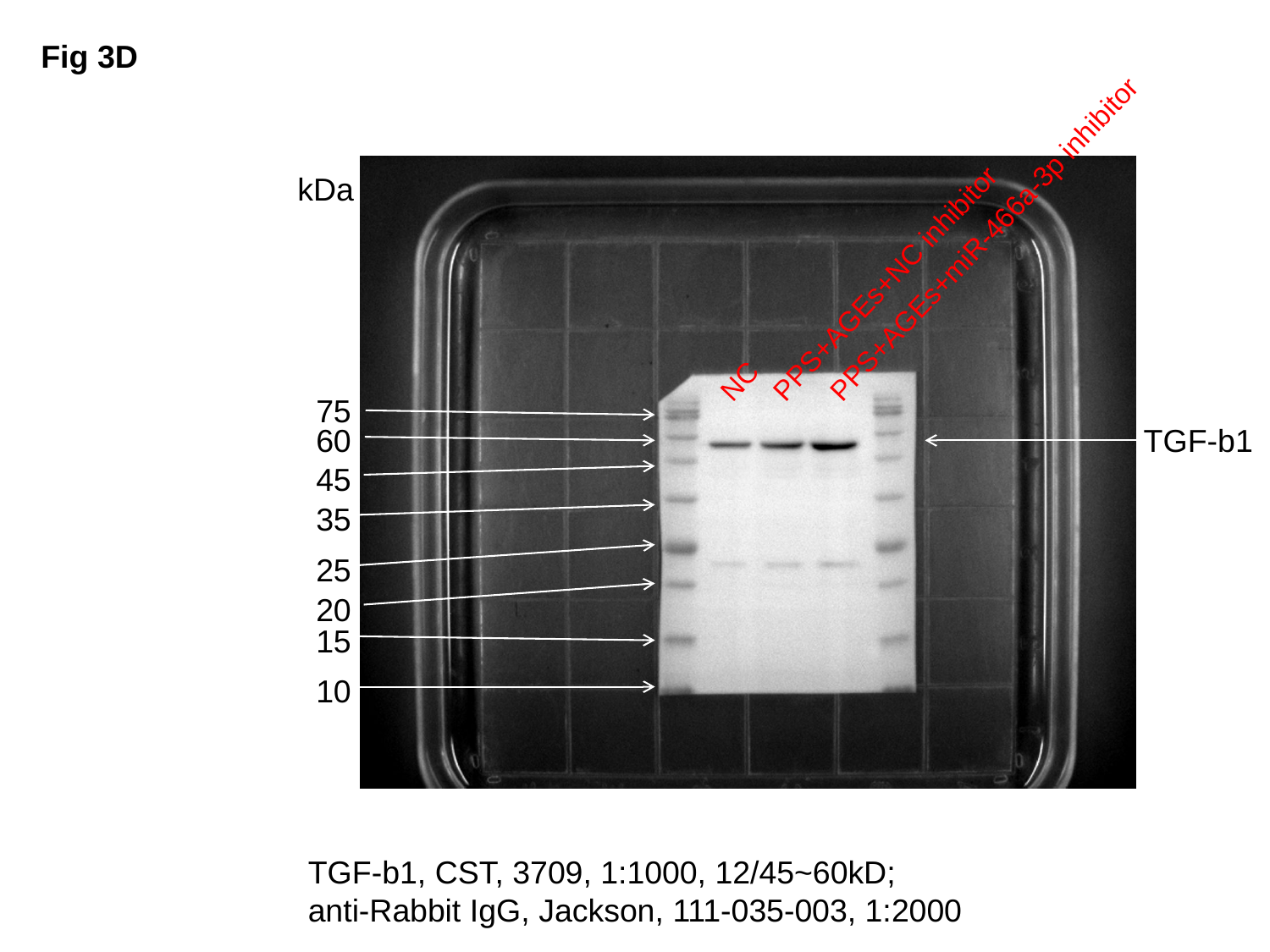

PPS+AGEs+miR-466a-3p inhibitor
PPS+AGEs+NC inhibitor
NC
Fig 3D
kDa
75
60
45
35
25
20
15
10
TGF-b1
TGF-b1, CST, 3709, 1:1000, 12/45~60kD;
anti-Rabbit IgG, Jackson, 111-035-003, 1:2000

## Slide 13
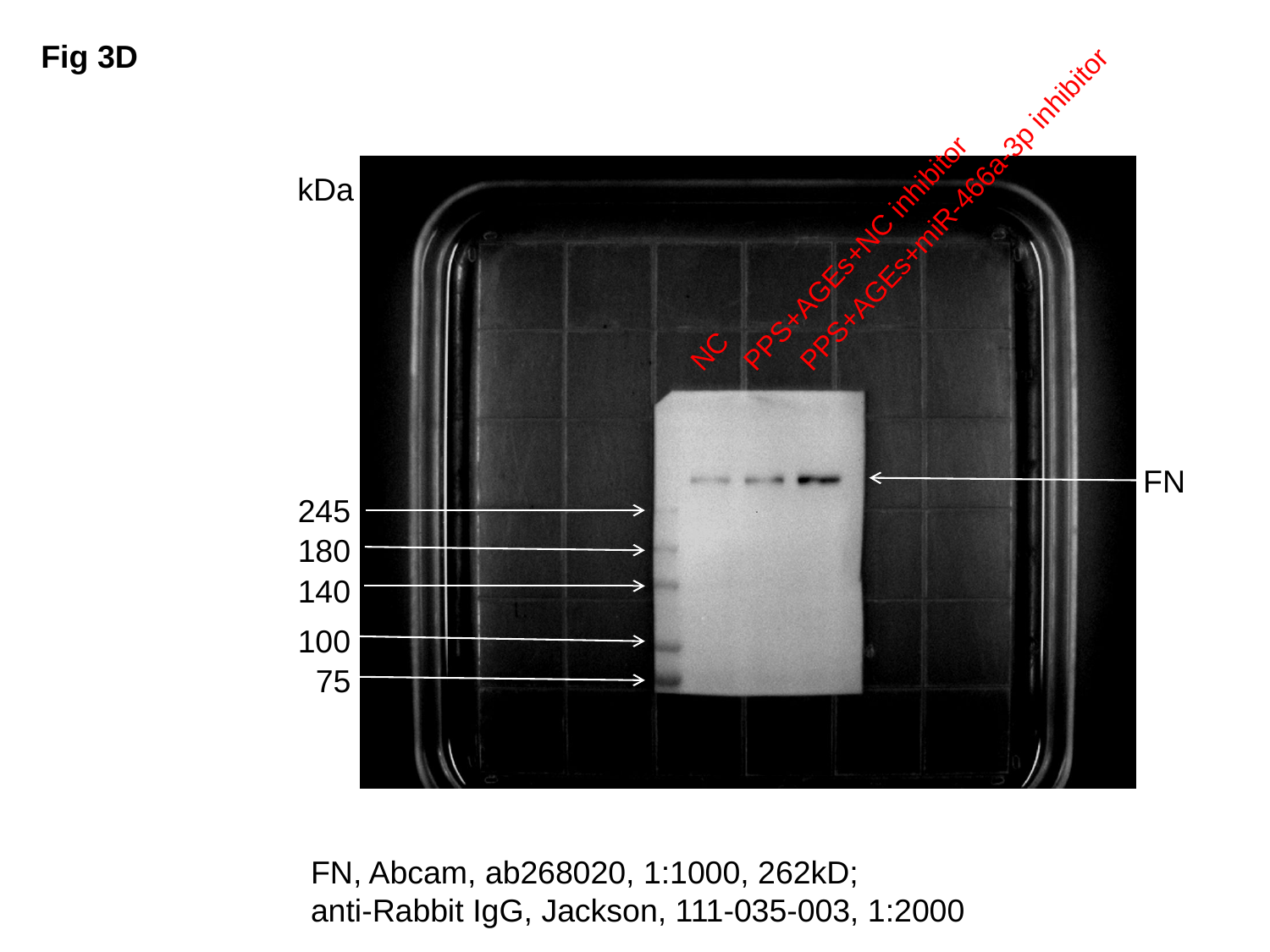

PPS+AGEs+miR-466a-3p inhibitor
PPS+AGEs+NC inhibitor
NC
Fig 3D
kDa
FN
245
180
140
100
75
FN, Abcam, ab268020, 1:1000, 262kD;
anti-Rabbit IgG, Jackson, 111-035-003, 1:2000

## Slide 14
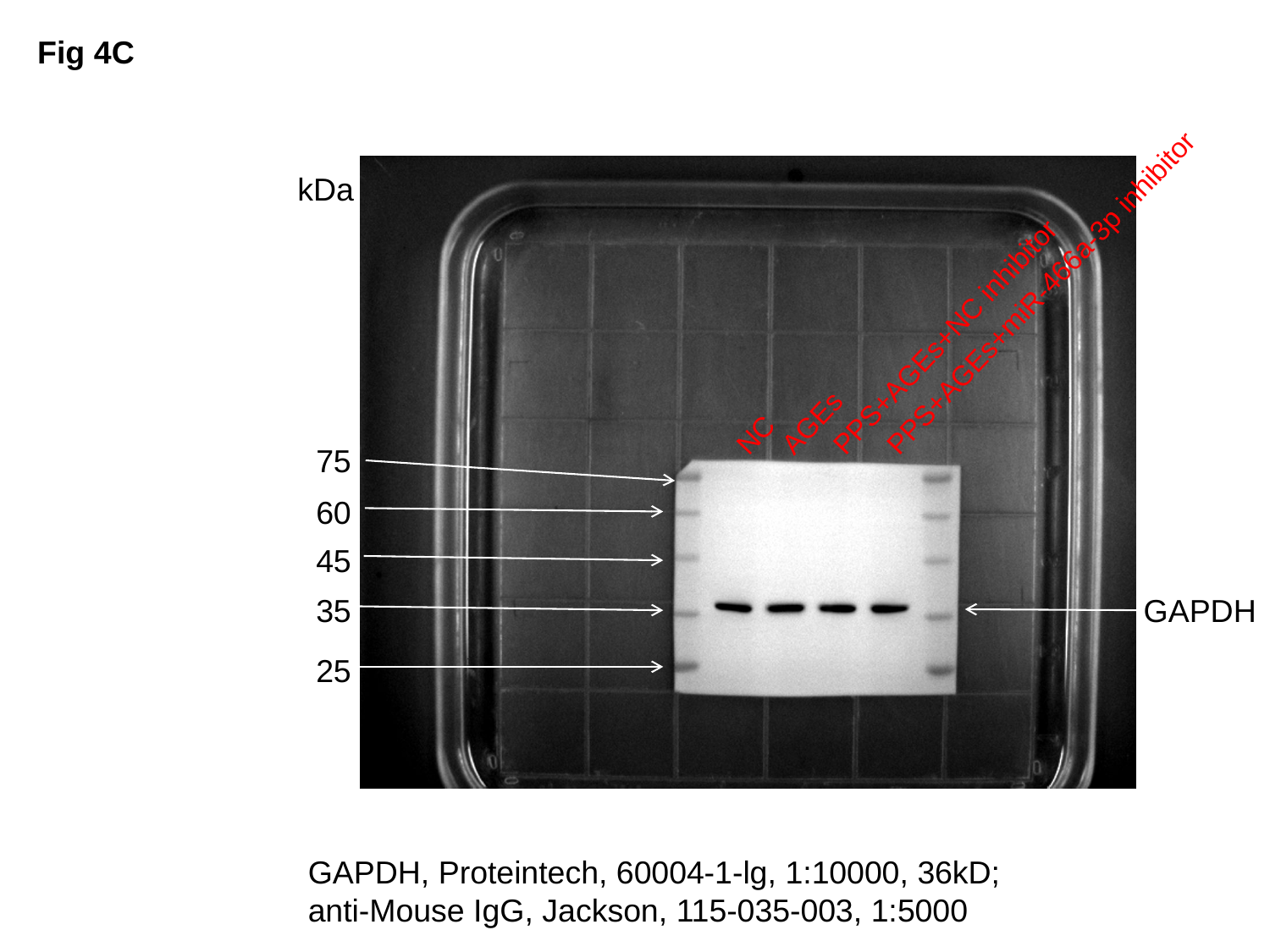

Fig 4C
PPS+AGEs+miR-466a-3p inhibitor
AGEs
PPS+AGEs+NC inhibitor
NC
kDa
75
60
45
35
25
GAPDH
GAPDH, Proteintech, 60004-1-lg, 1:10000, 36kD;
anti-Mouse IgG, Jackson, 115-035-003, 1:5000

## Slide 15
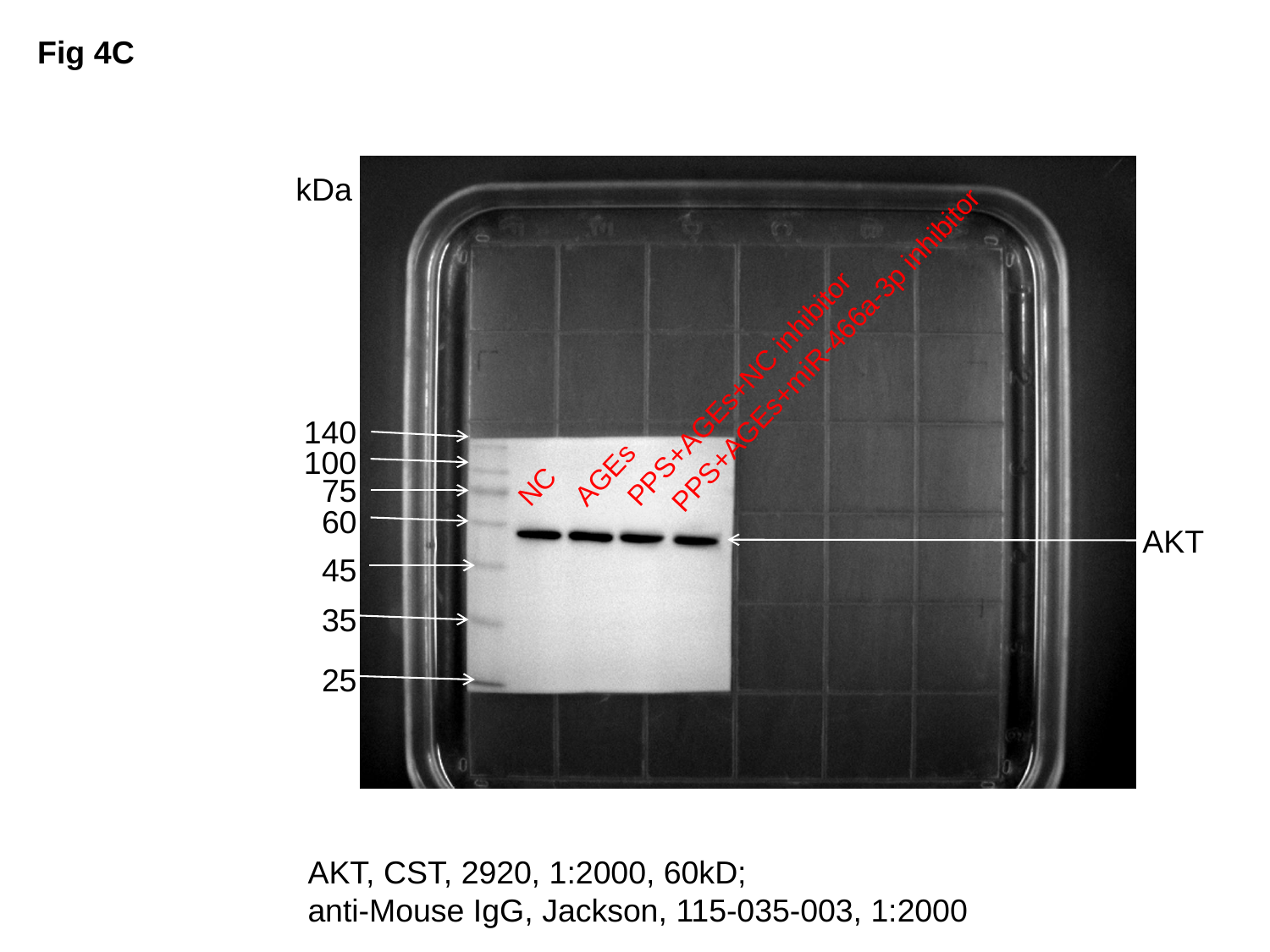

Fig 4C
PPS+AGEs+miR-466a-3p inhibitor
AGEs
PPS+AGEs+NC inhibitor
NC
kDa
140
100
75
60
45
35
25
AKT
AKT, CST, 2920, 1:2000, 60kD;
anti-Mouse IgG, Jackson, 115-035-003, 1:2000

## Slide 16
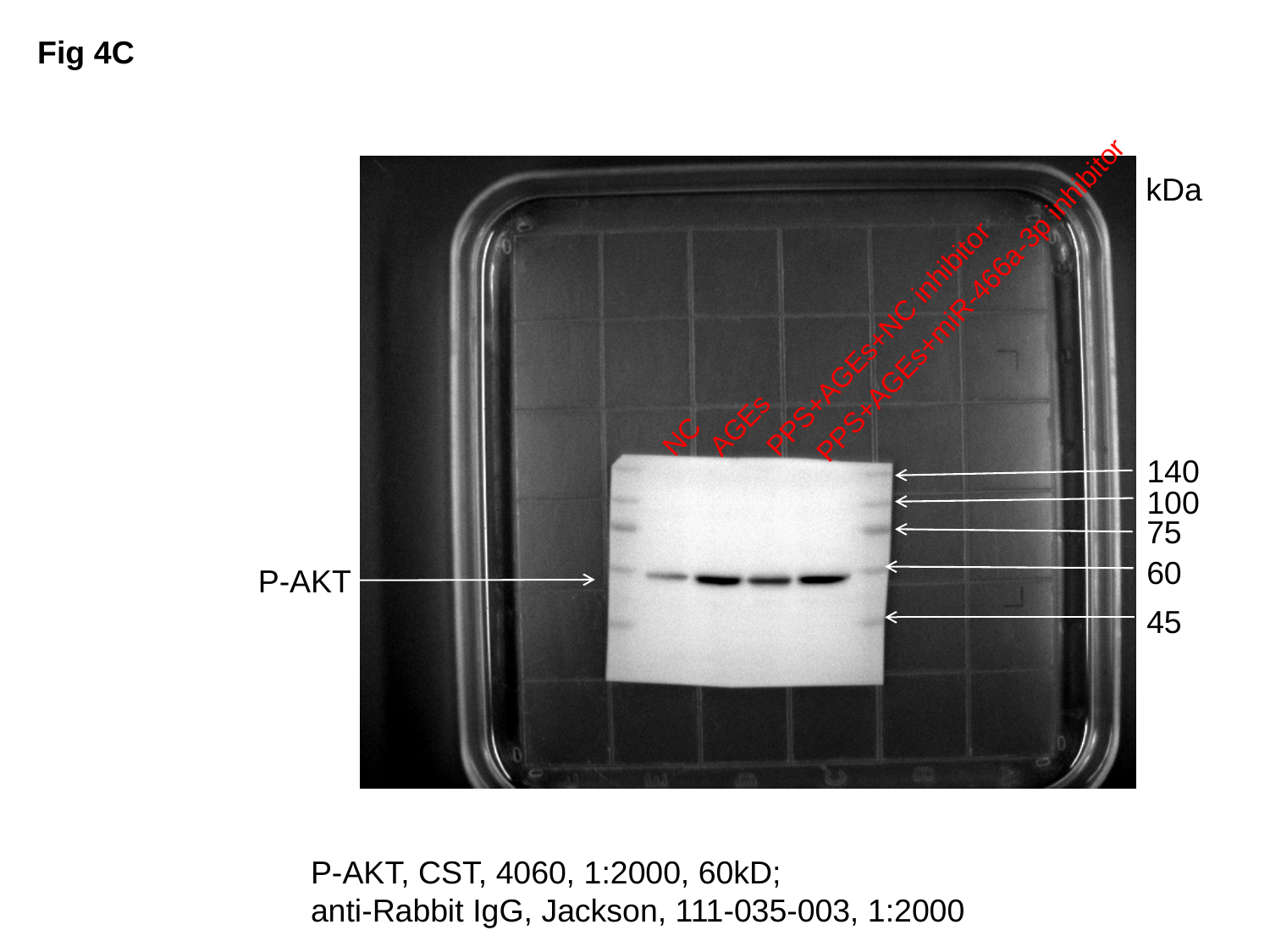

Fig 4C
PPS+AGEs+miR-466a-3p inhibitor
AGEs
PPS+AGEs+NC inhibitor
NC
kDa
140
100
75
60
45
P-AKT
P-AKT, CST, 4060, 1:2000, 60kD;
anti-Rabbit IgG, Jackson, 111-035-003, 1:2000

## Slide 17
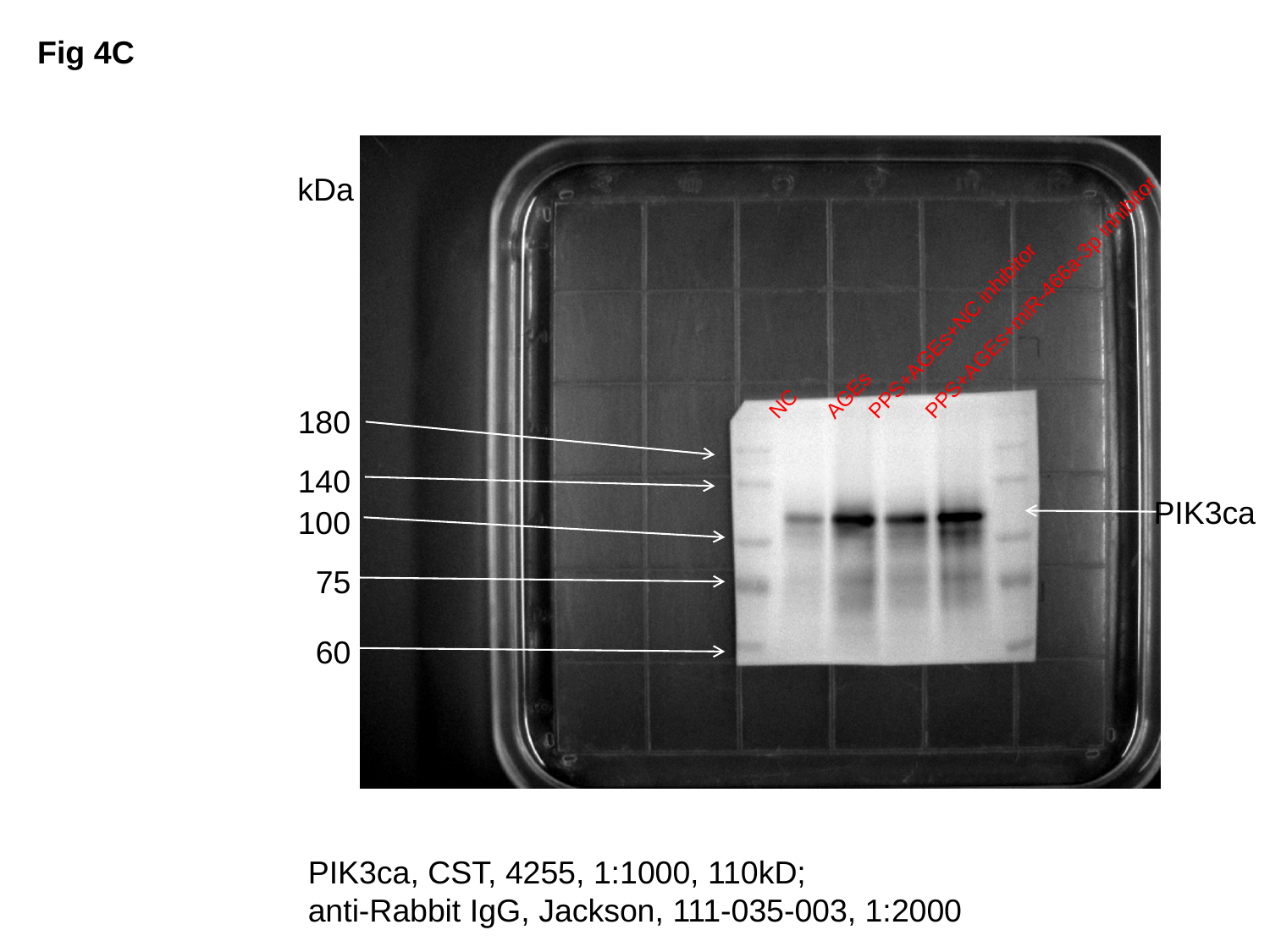

Fig 4C
PPS+AGEs+miR-466a-3p inhibitor
AGEs
PPS+AGEs+NC inhibitor
NC
kDa
180
140
100
75
60
PIK3ca
PIK3ca, CST, 4255, 1:1000, 110kD;
anti-Rabbit IgG, Jackson, 111-035-003, 1:2000

## Slide 18
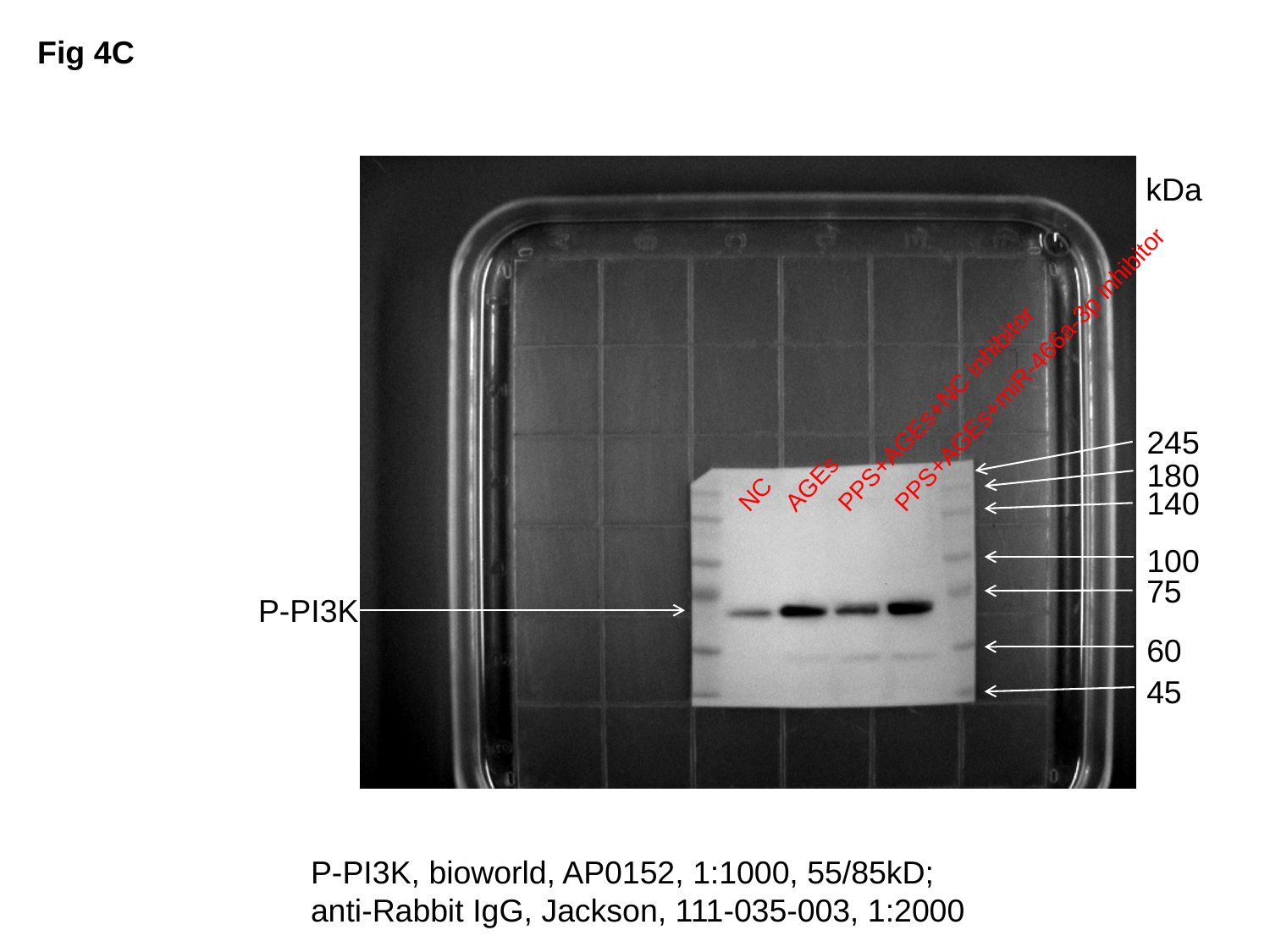

Fig 4C
PPS+AGEs+miR-466a-3p inhibitor
AGEs
PPS+AGEs+NC inhibitor
NC
kDa
245
180
140
100
75
60
45
P-PI3K
P-PI3K, bioworld, AP0152, 1:1000, 55/85kD;
anti-Rabbit IgG, Jackson, 111-035-003, 1:2000
