# Supplementary material for: Pentosan polysulfate ameliorates fibrosis and inflammation markers in SV40 MES13 cells by suppressing activation of PI3K/AKT pathway via miR-446a-3p
Source: BMC Nephrol. 2022 Mar 15;23:105. doi: 10.1186/s12882-022-02732-8 (PMC8925175; doi:10.1186/s12882-022-02732-8)
Supplement: Supplementary file 1 — Additional file 1. [file 12882_2022_2732_MOESM1_ESM.zip › Supplemental Figures.docx]

**Pentosan polysulfate ameliorates fibrosis and inflammation markers in SV40 MES13 cells by suppressing activation of PI3K/AKT pathway via miR-446a-3p**

Liangxiang Xiao, Anqun Chen, Qing Gao, Bo Xu, Xiaodan Guo, and Tianjun Guan

Department of Nephrology, Zhongshan Hospital of Xiamen University, School of Medicine, Xamen University, Xiamen, China

**Running title:** PPS protects against diabetic renal fibrosis

***Correspondence:**

Tianjun Guan

Department of Nephrology

Zhongshan Hospital Affiliated to Xiamen University

No 203, Hubin South Road, Siming district

Xiamen, Fujian,361004

Tel: 0592-2590261

Email: [tianjunguan@aliyun.com](mailto:tianjunguan@aliyun.com)

**Supplemental Figure**

**Figure S1. AGEs induced fibrosis and inflammation in SV40 MES13 cells.** (A) SV40 RES13 cells were treated with 200 μg/mL AGEs, and the cell viability was determined by MTT assay at 24, 48, and 72 h post-treatment. (B) Flow cytometry was used to determine the apoptotic rate of SV40 RES13 cells treated with AGEs for 24 h. (C) The protein levels of TFG- β1 and FN were determined by western blot analyses in SV40 RES13 cells treated with AGEs for 24 h. (D) The protein levels of IL-6 and TNFα were determined by ELISA assay in SV40 RES13 cells treated with AGEs for 24 h. All the experiments were performed in triplicate, and the results were analyzed by Student’s *t-*test (unpaired, two-tailed) and are expressed as mean ± SEM.

**Figure S2 Heatmap clustering analysis of the most significantly differentially expressed miRNAs induced by PPS co-treatment in AGEs-treated SV40 RES13 cells.**


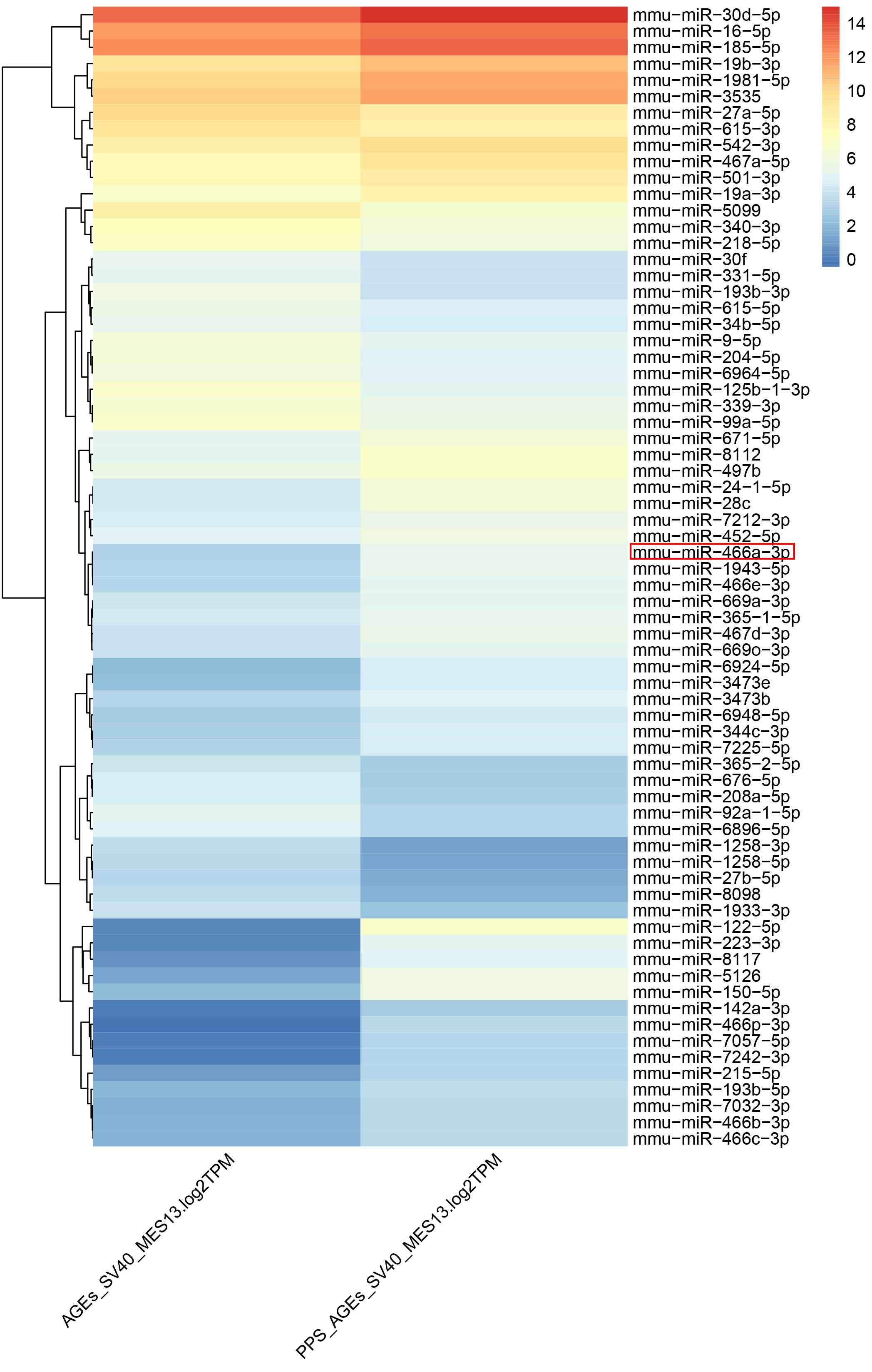


**Figure S3. Gene ontology (GO) analysis of potential targets of differentially expressed miRNAs.**  The top biological process, cellular component and molecular function classification terms for the AGEs vs. AGEs+PPS comparison. Red bars biological process, Green bars cellular component, Blue bars molecular function.

**
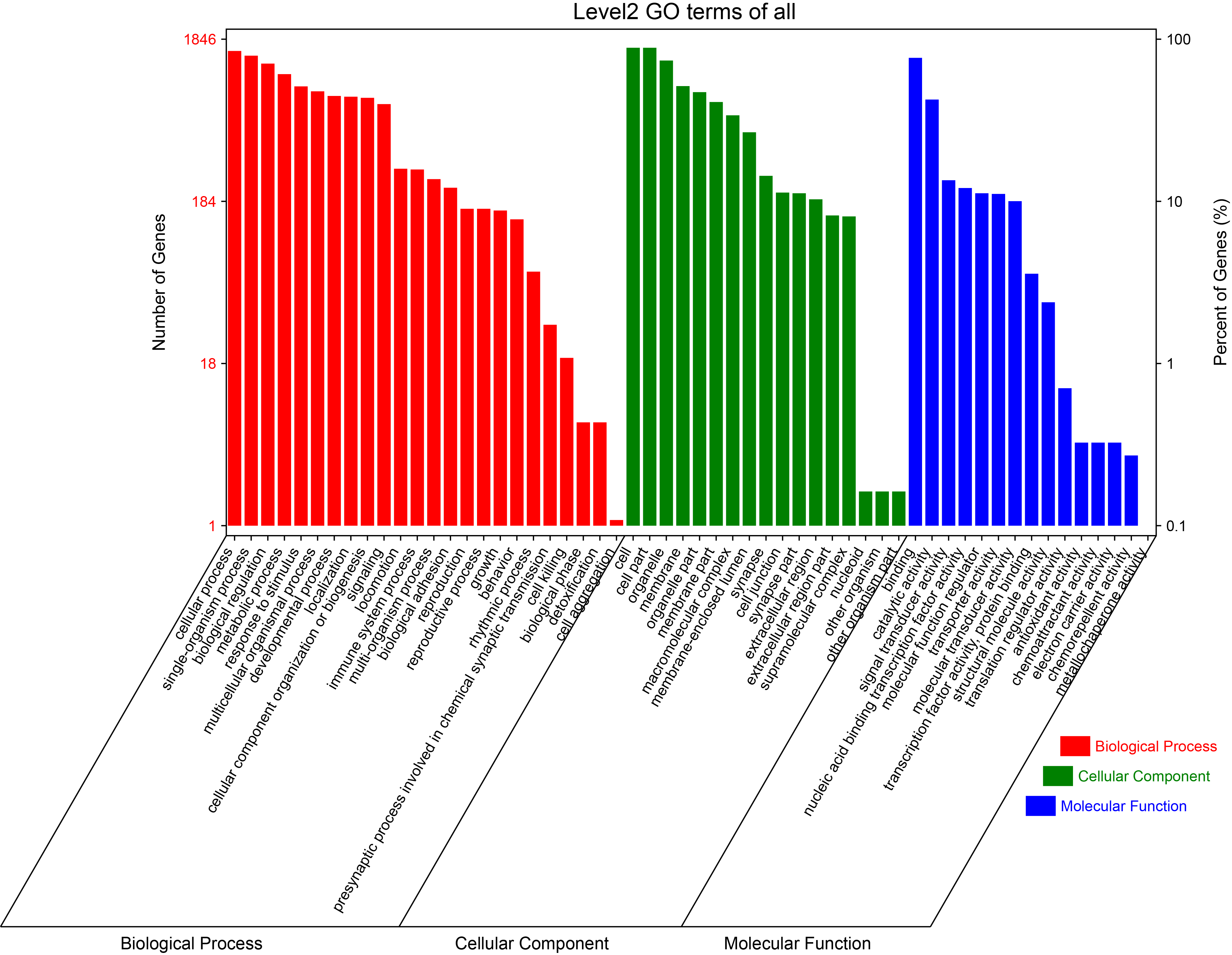
**
